# Comprehensive structural analysis of designed incomplete polypeptide chains of the replicase nonstructural protein 1 from the severe acute respiratory syndrome coronavirus

**DOI:** 10.1371/journal.pone.0182132

**Published:** 2017-07-27

**Authors:** Leonardo Vazquez, Luis Mauricio Trambaioli da Rocha e Lima, Marcius da Silva Almeida

**Affiliations:** 1 Instituto de Bioquímica Médica Leopoldo de Meis, Centro Nacional de Biologia Estrutural e Bioimagem (CENABIO), Universidade Federal do Rio de Janeiro, Rio de Janeiro, RJ, Brazil; 2 Faculdade de Farmácia, Universidade Federal do Rio de Janeiro, Rio de Janeiro, RJ, Brazil; INRA Centre de Jouy-en-Josas, FRANCE

## Abstract

The cotranslational folding is recognized as a very cooperative process that occurs after the nearly completion of the polypeptide sequence of a domain. Here we investigated the challenges faced by polypeptide segments of a non-vectorial β-barrel fold. Besides the biological interest behind the SARS coronavirus non-structural protein 1 (nsp1, 117 amino acids), this study model has two structural features that motivated its use in this work: 1- its recombinant production is dependent on the temperature, with greater solubility when expressed at low temperatures. This is an indication of the cotranslational guidance to the native protein conformation. 2- Conversely, nsp1 has a six-stranded, mixed parallel/antiparallel β-barrel with intricate long-range interactions, indicating it will need the full-length protein to fold properly. We used non-denaturing purification conditions that allowed the characterization of polypeptide chains of different lengths, mimicking the landscape of the cotranslational fold of a β-barrel, and avoiding the major technical hindrances of working with the nascent polypeptide bound to the ribosome. Our results showed partially folded states formed as soon as the amino acids of the second β-strand were present (55 amino acids). These partially folded states are different based on the length of polypeptide chain. The native α-helix (amino acids 24–37) was identified as a transient structure (~20–30% propensity). We identified the presence of regular secondary structure after the fourth native β-strand is present (89 amino acids), in parallel to the collapse to a non-native 3D structure. Interestingly the polypeptide sequences of the native strands β2, β3 and β4 have characteristics of α-helices. Our comprehensive analyses support the idea that incomplete polypeptide chains, such as the ones of nascent proteins much earlier than the end of the translation, adopt an abundance of specific transient folds, instead of disordered conformations.

## Introduction

Protein folding is linked to one of the principles of life: most of the functions in a cell are carried out by proteins that have to fold properly. Incorrectly, folded proteins have to be directed to proteasomal degradation; otherwise, they can lead to misfolded protein diseases, also known as proteopathias, which can culminate in prion disease, Alzheimer's disease, Parkinson's disease, amyloidosis, or cancer.

The process of translation, consisting of the ribosome molecular machinery with all the accessory molecular elements including mRNA, tRNAs, and chaperones, is the first process that calls attention when one wants to learn about protein folding. Ribosomes synthesize polypeptide chains, and during their synthesis, the nascent proteins can acquire its structure or only obtain in the end of the process.

The cotranslational fold has been studied essentially in parallel to the description of protein synthesis by the ribosome since the middle of the last century. Early observations made it clear that some nascent chains of β-galactosidase still bound to the ribosome are enzymatically active [1]. More direct evidence that a polypeptide still attached to the ribosome, can adopt its native fold came from the use of conformational antibodies raised against β-galactosidase and tailspike protein of phage P22. [2,3] Initial efforts to characterize the folding propensity of nascent polypeptide chains were performed with synthetic fragments of chymotrypsin inhibitor-2 from barley seeds, an alpha/beta protein with 64 amino acids that readily refolds by a two-state mechanism [4,5]. As measured by circular dichroism and ANS binding, the acquisition of secondary and tertiary structure starts simultaneously after completion of 80% of the polypeptide chain. Native-like tertiary structure, measured by intrinsic fluorescence of tryptophan, starts after the polypeptide chain is 95% complete. It is worth noting that even though the experimental conditions used in these studies were native-like, the purification process involved denaturing conditions, mainly because of the poor solubility of the polypeptides. Of special interest is the fact that the longest construct, with 63 residues, was recovered from bacterial inclusion bodies.

Important evidence that the process of folding occurs during the synthesis of a protein comes from the lower recovery of the native fold after the refolding of a full-length protein in comparison to a protein that has been synthesized in a vectorial fashion by the ribosome. The presence of chaperones is significant but not mandatory in the cellular milieu, and in fact is not sufficient to increase the *in vitro* folding recovery to the efficiency level of a polypeptide emerging from the ribosome. A well-studied example is the β-barrel of the green fluorescence protein (GFP), which becomes active after folding from a nascent polypeptide at a much higher efficiency than during *in vitro* refolding of its full-length polypeptide chain and independently of the cytosolic crowding or cellular chaperones [6].

In fact, the study of co-translational fold has greatly improved after the use of GFP. This protein has a very complex fold composed of a non-vectorial 11 stranded parallel/antiparallel β-barrel, and very importantly, its activity is the emission of fluorescence, which can be assayed in several experimental conditions, including inside the cells, while still bound to the ribosome. With this system, it was possible to identify that nascent GFP with 10 of the 11 β-strands outside the ribosome exit tunnel forms a non-native conformation that is remarkably stable [7]. More recently, the sequential compaction of sub-domains of the first nucleotide-binding domain from the cystic fibrosis transmembrane conductance regulator was detected using fluorescence resonance energy transfer [8].

Protein folding coupled to the polypeptide synthesis in the ribosome seems to be more important to some proteins than others. In fact, the speed of translation can dictate the folding competence. Sequences of rare codons in the mRNA, which slows down the translation at specific lengths of the protein, are concentrated at the end of the coding sequence for a protein domain [9–11]. Curiously, silent mutations can lead to the impairment of protein folding and function [12,13]. Likewise, it has been shown that slowing down the bacterial translation enhances the amount of natively folded heterologous eukaryotic proteins [14].

Nevertheless, the amount of data actually showing the presence of folding intermediates is almost inexistent for polypeptides representing the beginning of the translation, which includes either free designed polypeptides or nascent polypeptide chains still bound to the ribosome. Important technical hindrances account for this lack of data: First the nascent polypeptide chains have high tendency to aggregate; secondly, working with the large ribosomal complex with nascent polypeptide chains still bound to them causes an enormous signal interference when they are accessed by the current spectroscopic methods; finally, the scarce concentration of the staled ribosomes with the nascent polypeptide chains can be as low as in the range of nanomolar.

In this work, we want to address the folding of vectorial polypeptide intermediates designed from a complex 3D structure: the six-stranded mixed parallel/antiparallel β-barrel of nsp1 from the SARS coronavirus. This viral protein is expressed as a soluble protein in *Escherichia coli* only at lower temperatures, such as 18°C, indicating a strong dependence on cotranslational folding events in order to achieve its native fold. We show that designed nascent polypeptide chains of the nsp1 adopt intermediates with hydrophobic clusters and significant 3D compaction. These intermediates appear with 2 of the 6 β-strands and the α-helix, represented by the designed protein nsp1(13–66). Additionally, after the presence of 4 β-strands and the α-helix, in the designed protein nsp1(13–100), there is the observation of significant formation of secondary structure. These events could be detected since we designed a recombinant fusion protein that allowed the use of complementary biophysical techniques to identify folding intermediates earlier in the translation of a polypeptide than detected previously.

## Results

### Design of a new system to study cotranslational folding

The study of cotranslational folding involves the use of purified ribosome-bound nascent polypeptide chains or free C-terminally truncated proteins to mimic the growth of a polypeptide [15–23]. These ingenious experimental setups have been developed since the 60´s and they have provided fascinating clues regarding the nature of protein synthesis. However, these systems present three intrinsic experimental drawbacks: 1- the ribosomal-bound nascent chain is a complex of more than 2 MDa, precluding studies by many biophysical approaches, including NMR spectroscopic methods, to achieve high-resolution structural data; 2- the free C-terminally truncated chains are very unstable, and are frequently expressed in inclusion bodies. Because of that, they need to be denatured in order to allow them to be purified; 3- in both systems, the effective concentration of purified sample is limited (lower micromolar).

Here we develop an experimental setup that attaches GB1, the soluble and small (56 amino acids) immunoglobulin-binding domain of streptococcal protein G to the C-terminus of the truncated polypeptides, to mimic a growing chain appended to a structured scaffold [24]—Sebastian Hiller, personal communication.

Besides the intrinsic solubility and stability characteristics of the GB1 domain, it has a fast and very efficient fold, which avoids interactions with any N-terminal incomplete polypeptide chain during its synthesis by the ribosome [25].

We verified by the comparison of NMR chemical shifts that the GB1 domain does not have significant interactions with the N-terminal constructs (S1 Fig). This analysis relies on the combined chemical shift difference (Δδ) to indicate the level of conformational and chemical environment similarity among identical polypeptide sequences. The Δδ values were calculated according to the following equation [26,27]:

$$\Delta \delta ={\left\{[{\left({\Delta \delta }_{HN}\right)}^{2}+(0.14*{\left({\Delta \delta }_{N}\right)}^{2})]/2\right\}}^{\frac{1}{2}}$$

Where: Δδ is chemical shift difference, Δδ_HN_ are the values of chemical shift of the amidic hydrogens and Δδ_N_ are the values of chemical shift of the nitrogen.

The combined chemical shift differences were very small (Δδ < 0.1 ppm) throughout the polypeptide sequence, but there were significant differences in the chemical environment of the N-terminal residues Q2 and Y3, as well as residues A20 and V21, which are in a loop very close to the N-terminus of GB1. We infer that the alteration in the chemical shifts of these four residues was an effect of the presence of the spacer placed N-terminally to the GB1 domain. It is worth noting that the attached GB1 domain behaved like the native, well-folded globular domain, with well-defined secondary structures and high ^15^N{^1^H} NOEs (S2 Fig).

Additionally, to separate the N-terminal truncated domains of nsp1 constructs from the GB1 C-terminal domain during ribosomal synthesis we included a 20-residue spacer, which encompasses an extended loop of bovine beta-crystallin [28,29]. This linker is long enough to allow the N-terminal polypeptides to reach the surface of the ribosome during the beginning of the synthesis of the GB1 domain. This spacer provided a reasonably dynamic loop, according to our NMR data, which included narrow chemical shift dispersion (S2A Fig), close-to-zero secondary structure propensity calculated from the carbon chemical shifts (S2B Fig), and ^15^N{^1^H} NOE around ±0.2 (S2C Fig).

**Fig 1:**
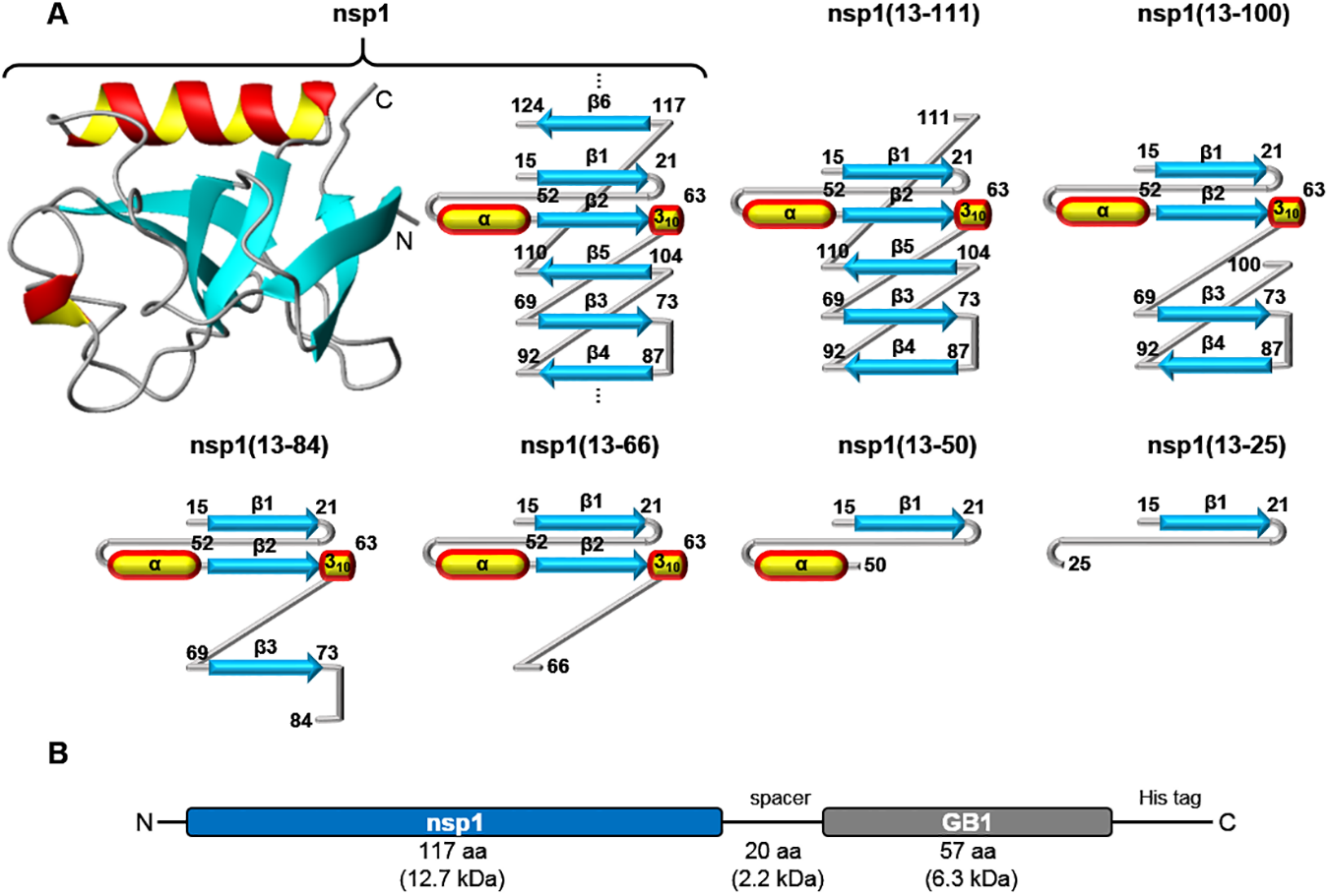
Scheme of the strategy used to study the co-translational folding of the nsp1 protein. (A) 3D structure and topology diagram of the nsp1 globular domain (without GB1 domain and spacer), and representation of the six hypothetical intermediates of its polypeptide growth during translation. The representation of the secondary structure elements are preserved for the purpose of visual guidance (B) Generic diagram of the designed fusion proteins. The blue segment contains one of the six polypeptide segments (nsp1(13–25), nsp1(13–50), nsp1(13–66), nsp1(13–84), nsp1(13–100) and nsp1(13–111)) of the nsp1 constructs detailed in (A).

As depicted in Fig 1A, we designed the nsp1 constructs so as to avoid truncating their secondary-structure elements. Constructs included six intermediates and the full-length chain of nsp1 protein. The nonvectorial nature of the β-barrel fold in nsp1 is evident in these representations. For instance, the intermediate nsp1(13–84) includes β-strand 3, which is not paired with β-strand 2 in the native fold, nsp1(13–111) includes β-strand 5, which is not paired with β-strand 4, and finally the full-length nsp1 globular domain includes β-strand 6, which is not paired with β-strand 5. The constructs ranged in size from 11 to 22 kDa and included, from the N- to the C-terminus (Fig 1B): An nsp1 construct; a spacer, designed to occupy the ribosome exit tunnel before the synthesis of the GB1 domain; a GB1 solubility domain; and a His-tag for purification. These constructs behaved similarly during their purification and provided us with stable samples for at least two weeks, with concentrations as high as 4 mM.

### Secondary structure begins to stabilize in construct nsp1(13–100)–two β-strands before the formation of the β-barrel

**Fig 2:**
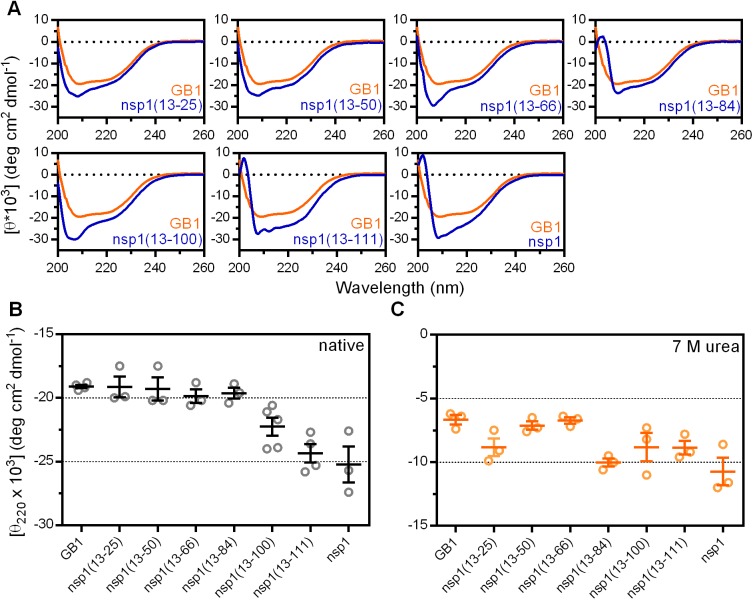
Circular dichroism spectroscopy analysis of fusion constructs designed to identify secondary structure formation. (A) Representative CD spectra of the fusion constructs at 30 μM in comparison to GB1 domain. Data were fit to a molar ellipticity curve using GraphPad Prism v.6. (B) Evolution of the ellipticity at 220 nm with the increment of the polypeptide chain. The mean and standard deviation of the independent measurements performed for different protein batches are indicated by bars. (C) Ellipticity at 220 nm evidencing the susceptibility of all constructs to 7 M urea.

We evaluated the overall content of secondary-structure elements by circular dichroism (CD) in the range of 200–260 nm (Fig 2A). The fusion construct containing the nsp1 domain as well as the six constructs containing incomplete polypeptide chains presented CD spectra distinct from the GB1 spectrum with an increase of negative ellipticity around 200–230 nm. An further increase in a negative band at 208 nm was noted especially for fusion constructs nsp1(13–25), nsp1(13–50), nsp1(13–66) and nsp1(13–100), which is correlated with the presence of random coil conformation [30]. The full-length nsp1 fusion construct displayed a CD spectrum very similar to the nsp1(13–111) truncated fusion construct, which includes a positive band in the range of 200–205 nm, typical of β-strand conformation in the presence of coil. This characteristic appeared even earlier, in the fusion construct nsp1(13–84), but was somehow obliterated in fusion construct nsp1(13–100). Even though the signal of secondary structure is significantly bigger in the intermediate fusion construct nsp1(13–100) than in the shorter fusion constructs, it does not resemble the CD spectrum of full-length nsp1. This most likely reflects an intermediate state of folding distinct from the other fusion constructs.

The transition for the formation of significant secondary-structure elements is better demonstrated in Fig 2B, since the negative ellipticity at 220 nm is commonly used to characterize the formation of both β-strands and α-helices. It is clear that the formation of secondary structure depends on the presence of strands β1–4 and helix α, represented by construct nsp1(13–100). The substantial emergence of stable secondary structure in this construct is related to the large increase in the amount of native long-range contacts after the presence of strand β4. The long-range contacts in the native structure of nsp1 are illustrated in the S3 Fig. The segment coding up to strand β3, represented by construct nsp1(13–84) has 43 long-range contacts, considering the native full-length nsp1 fold. In contrast, the construct nsp1(13–100) has 47 additional long-range contacts compared to construct nsp1(13–84). Furthermore, the native pairing among native strands β3 and β4 is maintained by 33 long-range contacts, which is more than three times the amount between β1 and β2 (9 contacts), β2 and β3 (7 contacts), or between each strand and the helix α (1–11 contacts).

The further increase of negative ellipticity in fusion construct nsp1(13–111) in comparison to nsp1(13–100) also follows the trend observed with the transition between construct nsp1(13–84) and nsp1(13–100), both for the increase in the 220 nm negative ellipticity and the amount of native long-range contacts. Considering the native structure, strand β5 has an extensive network of long-range contacts (36 contacts) with β3, and the construct nsp1(13–111) has 61 additional contacts compared to nsp1(13–100). Finally, the full-length protein forms a globular β-barrel fold, by the addition of the remaining 52 long-range contacts with β-strand 6.

In denaturing conditions (7 M urea), the fusion constructs and the GB1 construct lost most of their secondary structure signal, reaching an ellipticity level around -12 to -6 deg.cm^2^.dmol^-1^ (Fig 2C).

### Acquisition of tertiary structure in intermediate constructs of nsp1 after the acquisition of strand β2

**Fig 3:**
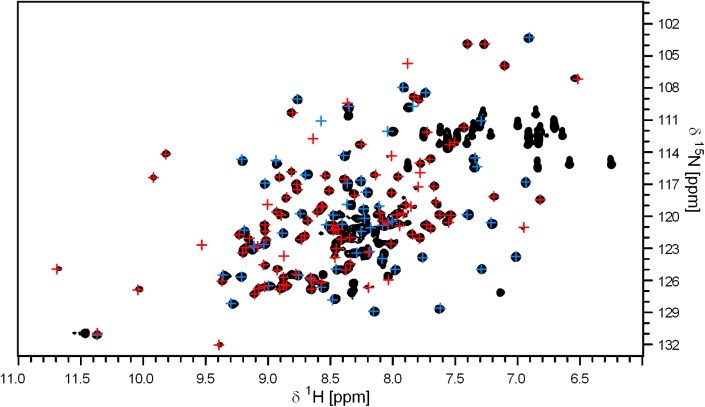
Identity of 3D structure of nsp1 and GB1 globular domains with the designed full-length fusion construct, as evidenced by the 2D [^1^H,^15^N]-HSQC spectra superposition. The H^N^ correlation signals of the full-length fusion construct, containing the nsp1 globular domain, a polypeptide spacer, the GB1 domain and a His-tag, is shown in black. The red crosses identify the signals belonging to the native isolated nsp1 globular domain. The blue crosses identify the signals belonging to the GB1 domain fused to a His-tag. The spectra were recorded at an ^1^H frequency of 600 MHz at 22°C.

We used NMR spectroscopy to characterize the tertiary structure of each construct. The 2D [^1^H,^15^N]-HSQC spectrum of the full-length fusion construct has a wide dispersion of chemical shifts, which resembles the spectra of the free nsp1 and the free GB1 domains (Fig 3). This data indicates that the nsp1 domain in the fusion construct has the same fold as the free domain and that it does not interact with the GB1 domain to a significant extent.

**Fig 4:**
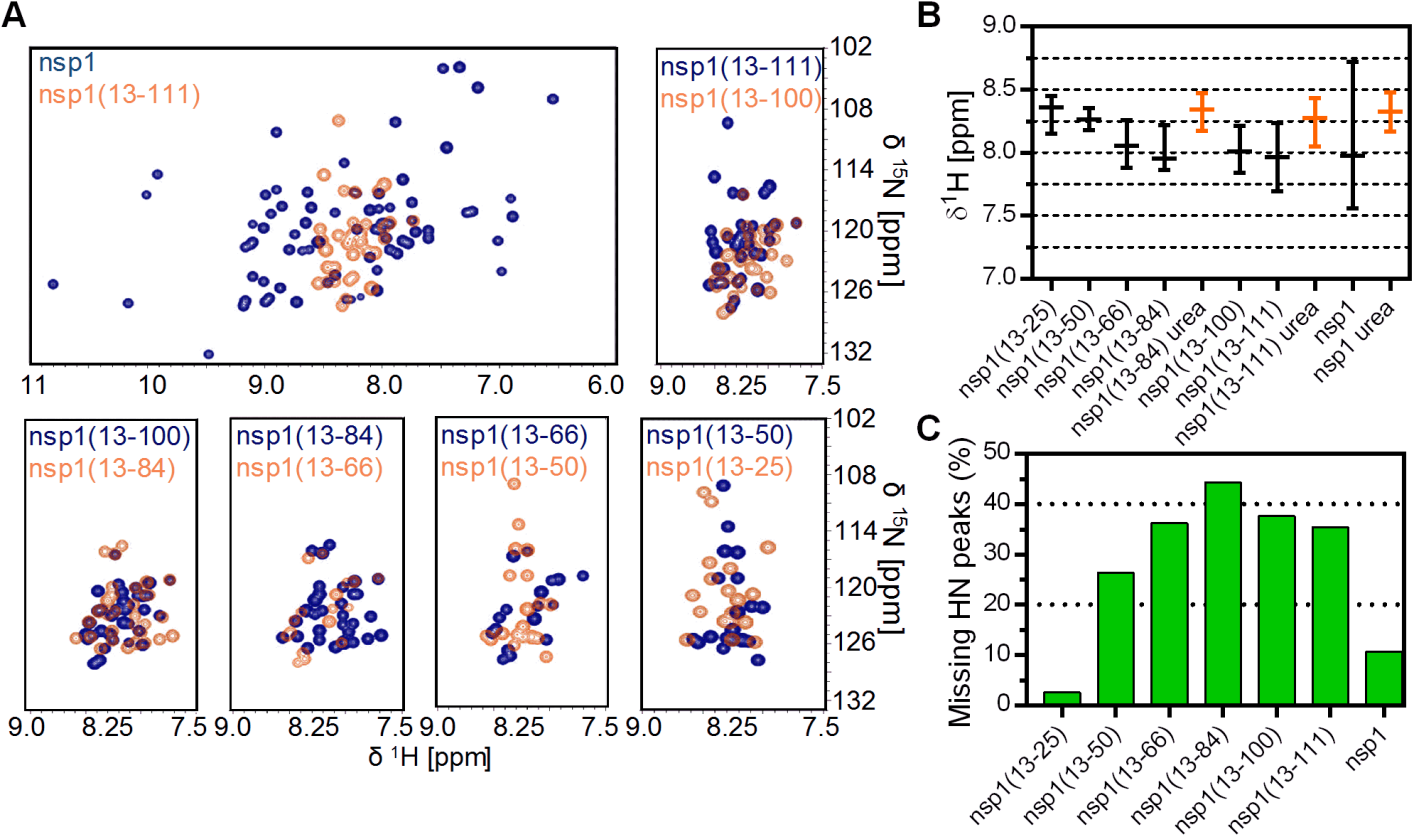
Tertiary structure analysis by two-dimensional NMR spectroscopy of each designed fusion protein. (A) 2D [^1^H,^15^N]-HSQC spectra of each designed fusion construct back-calculated with Cara v. 1.9.1.5 in order to present solely the backbone signals of nsp1. (B) ^1^H Chemical shift dispersion of each spectrum shown in (A). The bars represent the medians and the dispersions were calculated on the quartiles, which contain 75% of the signal in the amidic region. The data collected for selected samples in the presence of 7 M urea (orange bars) are included to represent denatured conformations. (C) Percentage of missing backbone HN signals in the 2D [^1^H,^15^N]-HSQC spectra of each designed fusion construct and GB1 domain alone.

The clear similarity of the resonance chemical shifts of the spacer and GB1 domain among all the fusion constructs, including the ones shown in S1 Fig, allowed us to identify the peaks belonging to each intermediate of the nsp1 domain as well as the globular full-length nsp1 domain. The spectrum showing exclusively the signals of the nsp1 segment in each designed fusion construct was then back-calculated and analyzed for ^1^H dispersion and number of peaks (Fig 4). The intermediate constructs have much lower dispersion than the well-folded, full-length domain, showing that the intermediates of nsp1 do not have a well-defined fold (Fig 4A). It is clear that the spectra of nsp1(13–25), nsp1(13–50) and nsp1(13–66) proteins do not overlap, indicating that these three fusion constructs have different conformations. Considering the longer fusion constructs, from nsp1(13–66) up to nsp1(13–111), there is considerable equivalence among the spectra; however, this can be merely a superposition of peaks from different amino acids and because of that cannot be considered fold similarity. Nevertheless, if we compare the median and the dispersion of signals of each fusion construct (Fig 4B), we see that the medians of the spectra from fusion construct nsp1(13–66) towards the full-length construct are very similar, around 8 ppm. On the other hand, the median signals of the two shortest fusion constructs, nsp1(13–25) and nsp1(13–50), are very similar to the urea-denatured samples nsp1(13–84), nsp1(13–111) and the full-length nsp1, all around 8.3–8.4 ppm. This indicates that the nsp1 polypeptides from nsp1(13–66) onward adopt different conformations than that of an unfolded structure. It is also noticeable that the dispersion of the fusion construct nsp1(13–111) is greater than the other intermediates, or urea-denatured samples, indicating that the nsp1 polypeptide segment of this sample adopted a more compact 3D fold.

The number of missing peaks in the 2D [^1^H,^15^N] correlation spectra (Fig 4C) is a straightforward and widely used tool to identify the quality of the fold for a sample, especially for relatively small proteins such as the fusion constructs used here (less than 22.2 kDa). The greater the number of missing peaks in 2D spectra, the more likely it is that the protein adopts an intermediate fold with intermediate conformational dynamics. It is worth noting that there are several examples in the literature showing that for mostly unfolded and highly dynamic proteins their 2D [^1^H,^15^N] correlation spectrum will present low dispersion but only a few missing peaks, because of superposition or intermediate dynamics of their 3D structures. This is in fact the case for the fusion construct nsp1(13–25), missing only 3% of its peaks. The number of missing peaks increases substantially to 44% with the length of the polypeptide chain from the fusion construct nsp1(13–50) to nsp1(13–84), and then decreases until it reaches approximately 11% in the full-length nsp1 domain. This increased percentage of missing peaks is an indication for the existence of folding states with intermediate conformational dynamics. The full-length nsp1 fusion protein has only a few missing peaks, indicating a well-folded 3D domain, which causes a wide dispersion of chemical shifts and allows straightforward identification of backbone HN signals.

A classic approach to identifying 3D structures with intermediate folding uses the fluorescence of bis-ANS dye [31,32]. A blue shift of the emission maximum and an increase of quantum yield of the bis-ANS fluorescence spectrum occur with decreasing dielectric constant of its surroundings, such as in the partition from aqueous solvent to protein hydrophobic microdomains. The binding of bis-ANS to proteins is dominated by hydrophobic interactions, and as such, its interaction with hydrophobic clusters and stable hydrophobic pockets, which are well-known indicators of intermediate folding structures, protein aggregation, or even specific active sites, such as nucleotide binding sites.

**Fig 5:**
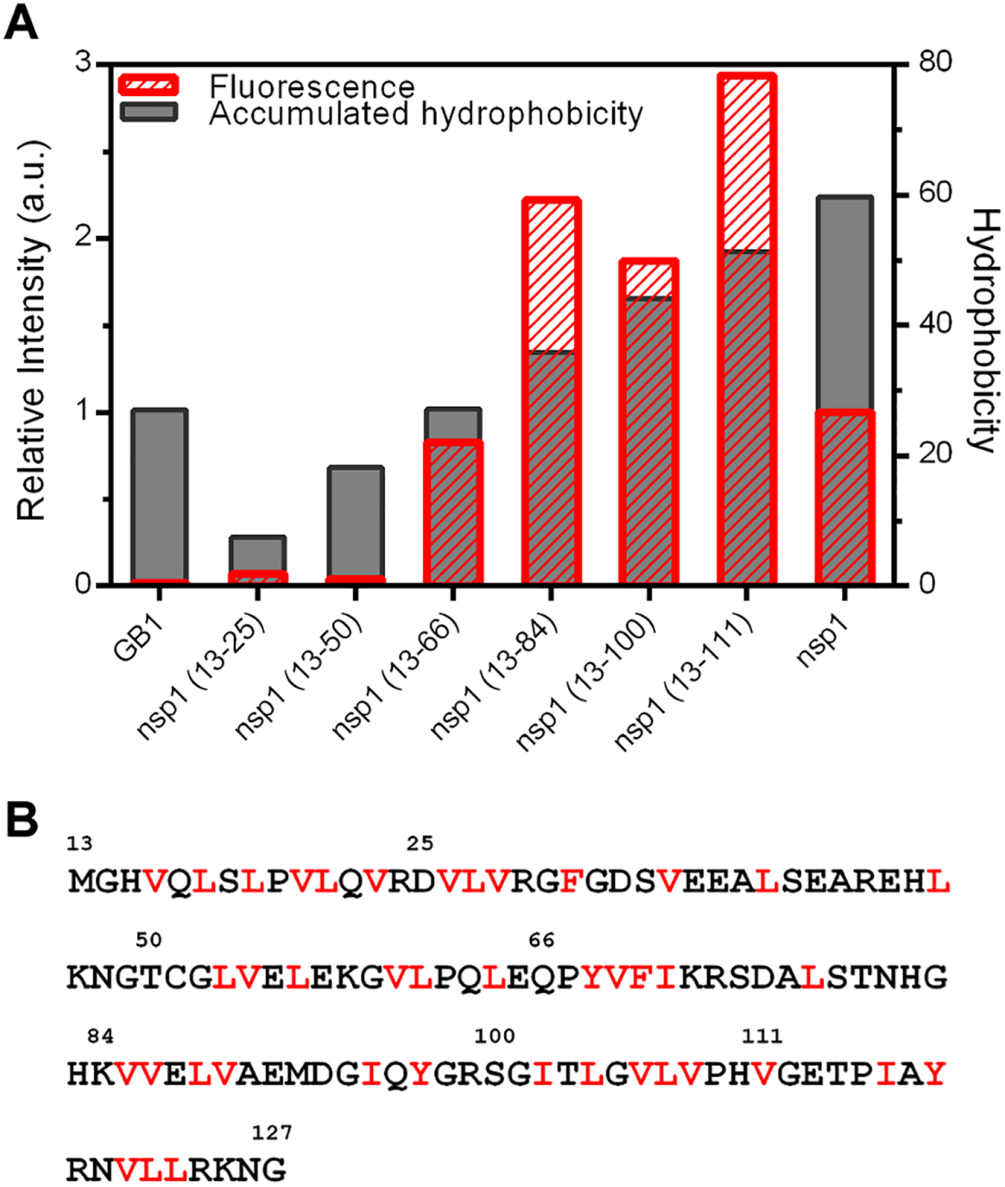
Identification of hydrophobic clusters in the designed fusion proteins. (A) Hydrophobic clusters were identified by the fluorescence intensity of bis-ANS (red bars), which was incubated with each sample, relative to the dye in the absence of protein (left axis). The accumulated hydrophobicity of GB1 domain fused to a His-tag and one of the incomplete polypeptide chains of the nsp1 protein as well as the globular domain of nsp1 are indicated as grey bars (right axis). (B) The amino acid sequence of the globular domain of nsp1 used in this study. The boundaries for each construct are indicated by numbers and hydrophobic residues (hydrophobicity > 0.8) are shown in red.

As shown in Fig 5, there is an increase in the bis-ANS fluorescence from fusion constructs nsp1(13–66) to nsp1(13–111). Significant extrinsic fluorescence is also observed in the full-length nsp1 sample, but only a residual signal is observed in the samples nsp1(13–25) and nsp1(13–50) as well as the GB1 domain alone. The most likely cause for the binding of bis-ANS to these samples is the formation of hydrophobic clusters in partially collapsed states or in oligomers. The increase of bis-ANS binding does not correlate linearly with the accumulated hydrophobicity [33] in each construct (primary sequence shown in Fig 5B), indicating that there are specific conformations forming hydrophobic clusters in the fusion constructs nsp1(13–66)- nsp1(13–111), which enable a very efficient binding of this hydrophobic dye. The fusion constructs present an average hydrophobicity of 0.51 ± 0.03 (accumulated hydrophobicity divided by the number of residues in each construct ± SD), ruling out the possibility of the existence of abnormally hydrophobic polypeptide chains that would have more affinity for bis-ANS. It is also worth mentioning that the urea-denatured samples do not bind significantly to bis-ANS dye (data not shown).

Nonetheless, the results from bis-ANS fluorescence indicate that after a specific polypeptide length, represented by the nsp1(13–66) fusion construct, there is a critical accumulation of hydrophobic residues that is sufficient to form hydrophobic clusters. Finally, there is a large decrease in accessible hydrophobic clusters in the full-length well-folded globular domain of nsp1, which is usually attributed to native hydrophobic collapses [34].

### Structural condensation after the acquisition of strand β4

**Fig 6:**
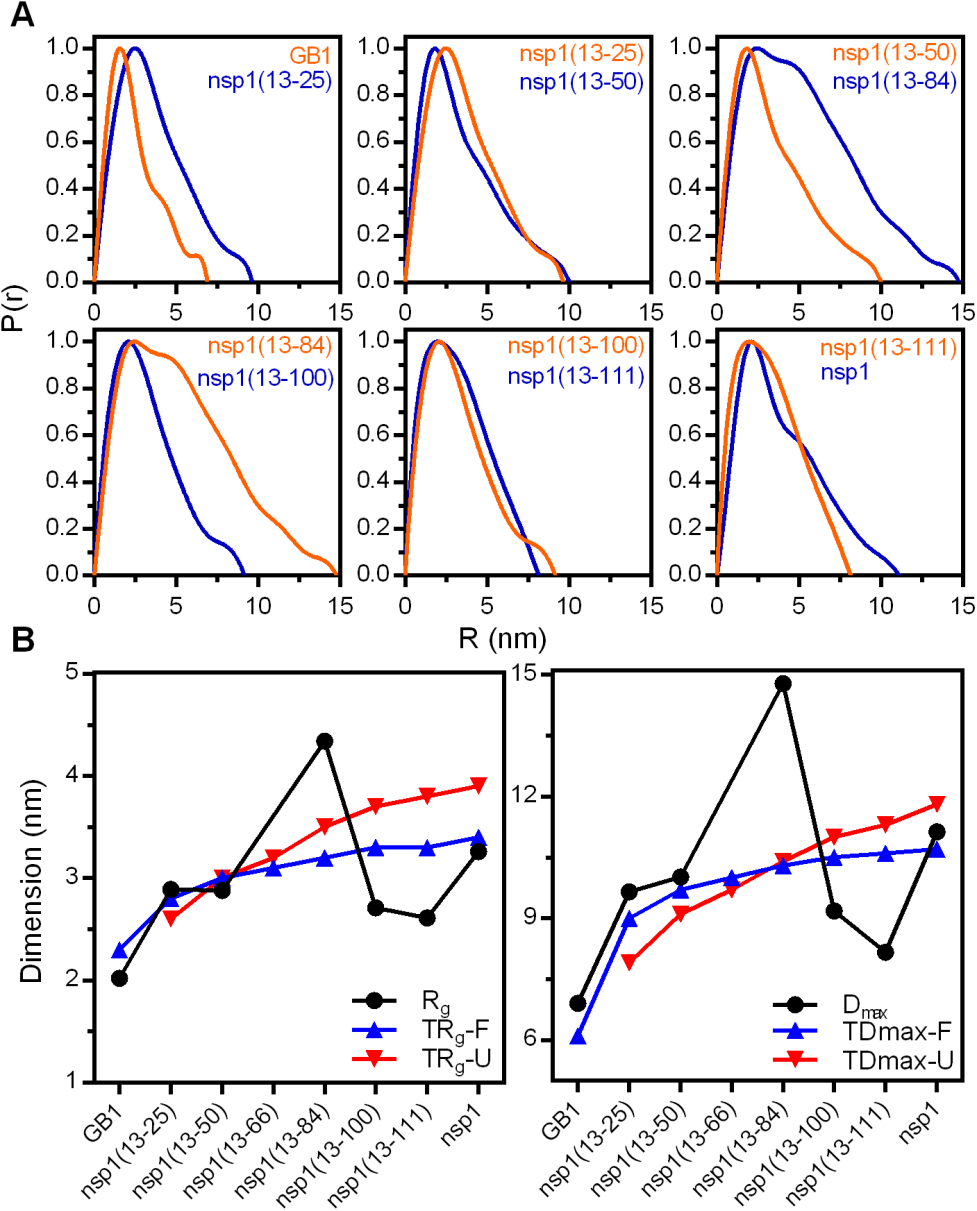
SAXS analysis of designed fusion proteins. (A) Distance distribution function of each designed fusion protein and GB1 domain fused to a His-tag. (B) Left panel—Plot of the Guinier radius of gyration (R_g_) and the calculated theoretical value of the hydrodynamic radius (TR_g_) of the designed fusion proteins considering the nsp1 segments in folded (blue) or unfolded (red) states. Right panel—Plot of the maximum distance (D_max_) and the calculated theoretical value of the maximum distance (TD_max_) of the designed fusion proteins considering the nsp1 segments in folded (blue) or unfolded (red) states.

In order to obtain deeper insight into the morphological features of the nsp1 fusion constructs in solution we collected small-angle x-ray scattering (SAXS) data [35,36], as shown here by the I_(q)_ scattering function. The GB1 domain alone behaved as a globular particle with R_g_ of 2 nm and D_max_ of about 6.8 nm (Fig 6), behaving as a compact structure as suggested by the Kratky curve (S4 Fig). From the Kratky plots we found indications of flexible elongated polypeptide segments in all designed fusion constructs, characterized by an increase of I_(q)_ × q^2^ plateauing at a given threshold. These data correlates with the other experimental observations presented here, which indicates the presence of a globular GB1 domain fused to distinct partially folded nsp1 polypeptide segments. The fusion construct nsp1(13–111) in particular has a consistent increase of the I_(q)_ × q^2^ along the q values (S4 Fig), indicating a high content of disordered structure. For the full-length nsp1 fusion construct, a sinusoidal function in the Kratky plot is observed, indicative of a compact, globular structure with no major intrinsically disordered components, compatible with the expected structure of two globular domains (GB1 and nsp1).

From the pair distance distribution P(r) data (Fig 6A), we calculated the R_g_ and D_max_ for each of the designed fusion proteins, as well as for GB1 domain alone, and compared with the hypothetical parameters for ideal folded and unfolded polypeptides of similar polypeptide chain length [37] (Fig 6B). In these calculations we used the equations Calc-Rh_FOLDED_ = (0.475 × Number of residues^0.29^) and Calc-Rh_UNFOLDED_ = (0.221 × Number of residues^0.57^) for folded or unfolded proteins, respectively (blue and red lines in Fig 6B). The R_g_ and D_max_ of GB1, as well as the designed fusion proteins nsp1(13–25), nsp1(13–50) and full-length nsp1, follows the expected dependence of dimensional parameter upon the chain length. A pronounced upper-deviation from the theoretical function is evident for the fusion construct nsp1(13–84), most likely identifying the expansion of the mean occupied conformational space. The fusion constructs nsp1(13–100), nsp1(13–111) show a reduction in R_g_ and D_max_ compared to the theoretical and to the other fusion constructs, indicating protein condensation upon crossing a given threshold between chain length as of the completion of the segment nsp1(13–84)-nsp1(13–100). We assume that this behavior may be attributed to additional structural stability for the previous strands (β1-β3).

Moreover, according to the plots of the distance distribution function P(r) (Fig 6A), all the fusion constructs are clearly represented by a small globular domain with a radius of approximately 3 nm, most likely from the GB1 domain, elongated up to approximately 10 nm (or 15 nm in the nsp1(13–84) protein), which we interpret as the contribution of the nsp1 chains, the spacer and the His-tag. The full-length nsp1 fusion construct presents two clearly defined minor radii of approximately 3 nm and 6 nm, which fit with the presence of the GB1 and nsp1 globular domains.

### Propensity to form α-helix in incomplete polypeptide segments that adopt native β-strands

With the exception of constructs nsp1(13–25) and nsp1(13–50), the intermediates of nsp1 have consistent indications for the formation of hydrophobic clusters and, after construct nsp1(13–100), the presence of defined secondary structure. In order to characterize the structure of the intermediates of nsp1 at atomic level we performed sequence-specific resonance assignment, which afforded us a more detailed view on the conformation of selected constructs: nsp1(13–25)–should not have any secondary structure; nsp1(13–50)–contains the polypeptide sequence that codes helix α, a secondary-structure element that is well known to have a significantly higher intrinsic folding propensity; nsp1(13–100) emerge as the smallest sample to show evidence for conformational collapse (Fig 6B), and might represent an important intermediate with two β sheets (see Fig 1A).

We were able to assign most of the backbone resonances for these three samples regardless of significant signal overlaps, typical for samples with non-globular 3D structure. The most representative exception is the segment from residues 74–87 in fusion construct nsp1(13–100). This segment corresponds to the first half of the most flexible loop in the native structure, which connects native strands β3 and β4. The signals belonging to this segment were absent in the spectra collected, most likely due to intermediate polypeptide dynamics, in the range of milliseconds. Polypeptide chain motions in this range are usually represented by conformational exchange of loops that are flexible but not thermally disordered. This intermediate dynamics causes NMR line broadening due to the sampling of different chemical environments in a ratio close to the difference in chemical shift of each state, a fundamental NMR property observed since the first studies of protein conformation [38].

With the ^13^C^α^, ^13^C^β^ and H^α^ chemical shifts we calculated the secondary-structure propensity (SSP) for the three fusion constructs nsp1(13–25), nsp1(13–50), nsp1(13–100) and the globular domain of nsp1. The SSP indicates the propensity ranging from -1 to 1 to adopt backbone conformations typical for extended β-strands or helical structures, respectively, according to the effect of these conformations on the chemical shifts of backbone atoms,[39–41]

**Fig 7:**
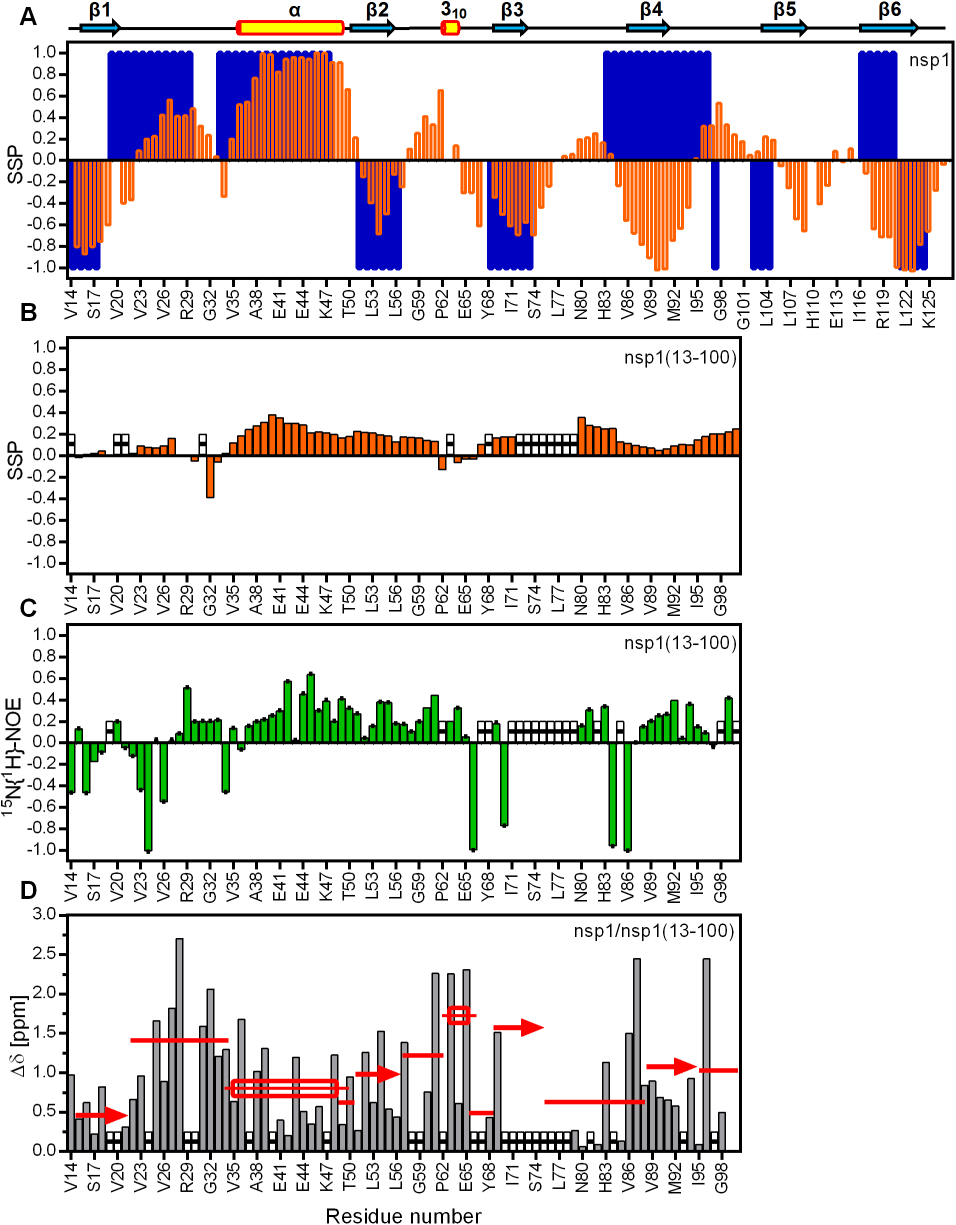
Per-residue secondary structure propensity and dynamics of the nsp1(13–100) designed fusion protein and the full-length globular domain of nsp1. (A) The orange bars represent the secondary structure propensity (SSP), and the blue bars are the values predicted on the basis of the nearest amino-acid residue neighbor in the sequence using the online server: Advanced Protein Secondary Structure Prediction Server (APSSP). Positive values identify helical conformation, while negative represent extended conformation such as β-strands. The secondary structure elements extracted from the 3D solution structure of nsp1 are identified across the top. (B) Secondary structure propensity of the nsp1(13–100) designed fusion protein. The white hatched bars identify positions without data. (C) Heteronuclear ^15^N{^1^H}-NOE values of the nsp1(13–100) designed fusion protein (green bars). Positive values represent less dynamic to rigid residues, while close-to-zero and negative values identify highly dynamic residues. The standard errors are indicated. (D) Differences in the H^N^ backbone chemical shifts (Δδ) of the nsp1(13–100) designed fusion protein compared to the full-length nsp1 protein (grey bars). The average Δδ for each secondary structure segment as well as their connecting loops are represented in red.

As shown in Fig 7, with the SSP algorithm it was possible to define very well the secondary-structure elements of the folded globular domain of nsp1 (orange bars in the uppermost graph). Moreover, most of the native secondary structures can be predicted solely based on amino-acid composition (blue bars in the Fig 7A), with striking exceptions of β-strand 4 and the first half of β-strand 6.

The intermediate nsp1(13–100) presents mainly a propensity to form helical conformations, under non-denaturing buffer. One segment with significant helical propensity is in a region that includes the native helix α, but also encompassing strand β2. This helical propensity is present in the same segment of the nsp1(13–50) intermediate, while the nsp1(13–25) construct, which has only the sequence coding for the first β-strand and part of the first loop, has propensities close to zero (data not shown).

One quite intriguing behavior of the nsp1(13–100) intermediate is that none of the native β-strands have propensity to be in extended conformation. Furthermore, there is the presence of helical propensity for the polypeptide segments that form the native strands β2 and β3, peaking at 0.25, and to a lesser extent strand β4, peaking at 0.15. To the best of our knowledge, this effect has not been previously detected in any intermediate of nascent polypeptide chains. Interestingly, the opposite propensity was shown for sperm-whale apomyoglobin, an all-α-helical protein [42]. At relatively short lengths of the apomyoglobin, a predominantly non-native β-sheet is present, but as chain length increases, α-helical conformation progressively takes over.

We also evaluated the polypeptide backbone dynamics by measuring ^15^N{^1^H} NOEs, which gives information on the motion of the H^N^ moiety for individual residues in a protein [43]. The 2D ^15^N{^1^H}-NOE experiment is very useful for characterizing the backbone H^N^ dynamics at pico to nanosecond timescales. NOEs with intensity of around 0.8, identify rigid polypeptide segments, and are usually found in secondary structure elements and folded core of proteins. Residues that undergo fast picosecond motion are identified by negative or NOEs with decreased intensity (minimum intensity at around -3.5).

The ^15^N{^1^H}-NOE profile for nsp1(13–100) indicated the existence of flexible, disordered residues (ps-ns time scale) in its N-terminus up to residue V26, residue V35, and a few residues around strand β3 and the end of the loop connecting this strand to strand β4. Residues with apparent lower flexibility (NOE peaking at ~0.6) are clustered in the polypeptide segments that correspond to the native helix α, 3_10_ helix and strands β2 and β4.

We collected 3D NOESY data for the three samples mentioned in this subsection, and noticed that they are very sparsely populated with homonuclear NOEs within the nsp1 polypeptide segments, showing almost exclusively the expected sequential *d*_*αN*_ and *d*_*NN*_ (data not shown). It is worth noting that the GB1 globular domain in these fusion constructs exhibited a very reasonable number of NOEs, compatible with the number observed for the isolated domain (data not shown). The paucity of NOEs, especially long-range ones, is a strong indication for the lack of stable tertiary structure within the studied nsp1 segments, but does not exclude the existence of partially folded species in these samples, which in fact corroborate with the data presented here.

Finally, we performed a pairwise comparison of the backbone H^N^ chemical shifts of nsp1(13–25) with nsp1(13–50), nsp1(13–50) with nsp1(13–100), and nsp1(13–100) with the full-length globular nsp1. As noted in the Fig 7D, the chemical shifts of nsp1(13–100) are quite distinct from those of the full-length globular domain, evidence that the conformations of these constructs are not alike. Nonetheless, it is noticeable that the loop connecting strand β1 and helix α, as well as the polypeptide sequence surrounding helix 3_10_, have the highest Δδ, suggesting that these are the regions that suffer the greatest conformational change, from nsp1(13–100) to full-length globular nsp1. The smaller Δδ in strand β1 and helix α indicate that these two elements are already in a chemical environment more similar to the hydrophobic core of stably folded nsp1.

As revealed previously by SSP, the Δδ analysis also indicated that the conformation of nsp1(13–25) is very similar to that of its corresponding sequence in nsp1(13–50) and nsp1(13–100), since the Δδ values between nsp1(13–25) and nsp1(13–50) and between nsp1(13–50) and nsp1(13–100) are below 0.25, with the sole exception of residues 22 and 25 in nsp1(13–25) (averaging Δδ~0.4) and 23, 28 and 45 in nsp1(13–50) (averaging Δδ~0.9) (data not shown).

## Conclusions

Even though it is intuitive to expect the formation of a β-barrel only after the translation of the last β-strand, there is the possibility of the existence of intermediates on the cotranslational folding of nsp1, including (shown in Fig 1): 1- the presence of the α-helix, first seen in the construct nsp1(13–50); 2- the existence of one β-sheet formed by β-strand 1 and β-strand 2, in constructs nsp1(13–66), nsp1(13–84) and nsp1(13–100); 3- the formation of a second β-sheet with β-strands 3 and 4 in the construct nsp1(13–100); 4- the union of the two intermediate, double-stranded β-sheets by the translation of β-strand 5 in construct nsp1(13–111).

However, in spite of nsp1(13–50) having an α-helical propensity in a very similar polypeptide segment as the native helix α, our data support a different scenario. First, there is no evidence for the formation of any intrinsic β-sheet intermediate. In its place, at fusion construct nsp1(13–100) there is a significant α-helical propensity for the polypeptide segments that form the native β-strands 2 and 3, and to a lesser extent β4. The detection of non-native folding intermediates with α-helix propensity within segments of native β-strands has been described in the literature for full-length β-lactoglobulin and canine milk lysozyme [44–47], and these elements are considered intermediates on the protein-folding pathway.

The formation of the native α-helix must be aided by the long-range contacts involving at least strands β1-β5 of nsp1, since there is no stabilization of this secondary structure up to the fusion construct nsp1(13–100), but probably in nsp1(13–111), which has the most similar CD spectra to the full-length fusion construct, at the same time very distinguishable from the other fusion constructs (Fig 2). In parallel, the abundance of long-range contacts indicates that the α-helix is important for the stabilization of the β-barrel. The fact that this helix has the highest propensity among all the secondary-structure elements of nsp1 that are formed after the intermediate nsp1(13–50) corroborates this hypothesis. We envisage testing this by comparing a construct lacking this helix with a synthetic polypeptide encoding the native α-helix.

**Fig 8:**
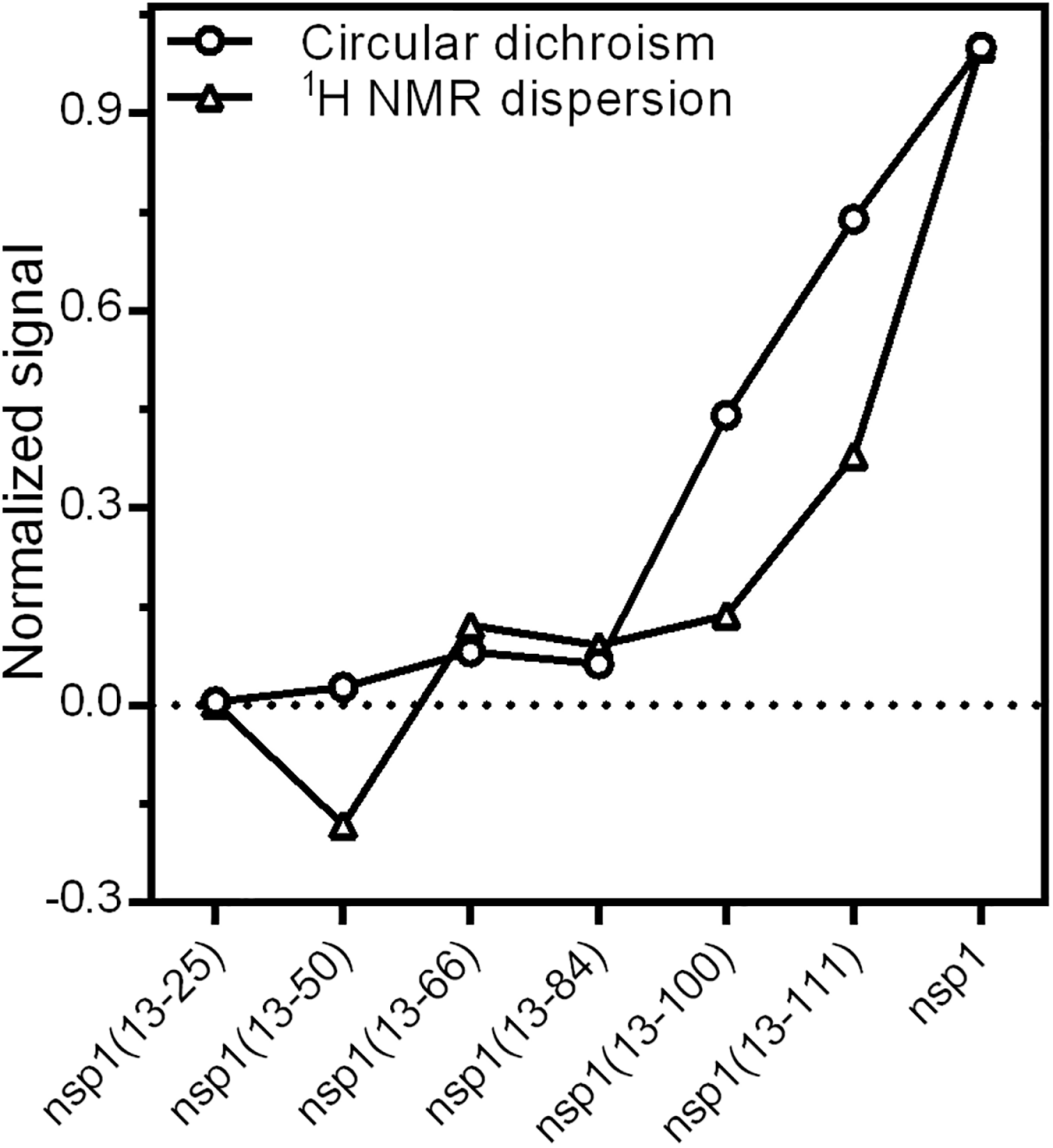
Normalized content of secondary (circles) and tertiary (triangles) structures. The normalized signals were experimentally determined by CD (Fig 2B) or NMR spectroscopy (Fig 4B) and plotted together to highlight these events within the designed fusion polypeptides. Signals are normalized to the highest value (full-length nsp1).

It is clear that the incomplete polypeptides of nsp1 do not adopt stable conformations. This is common sense with regard to the cotranslational fold, including for proteins that have been studied by the use of truncated polypeptides, such as barnase (RNase from *Bacillus amyloliquefacies*), chymotrypsin inhibitor 2 (CI2), staphylococcal nuclease and sperm-whale apomyoglobin [48–51]. In polypeptide chains nearing completion (>95% of the final length), these authors identified compact structures with long-range interactions, perhaps non-native, but lacking stable secondary structures. It is worth mentioning that the purification of these truncated proteins involved chemical denaturation; we infer that the results were obtained with refolded samples, which is an additional variable in the experimental setup, not present in the natural cellular environment. Our experimental results indicate a different scenario, where intermediate polypeptide lengths of nsp1, represented by designed proteins nsp1(13–100) and nsp1(13–111), start to adopt stable secondary structure and then tertiary structure before the completion of the polypeptide chain, observed for the designed protein nsp1(13–111) (Fig 8).

Nevertheless, we identified the formation of dynamic hydrophobic clusters, which are distinct from the chemically denatured samples. This effect is also observed in the full-length globular domain of nsp1 and reaches a peak with the fusion construct nsp1(13–111) (Fig 5), which acquires tertiary structure according to our NMR data (Fig 4), albeit still with substantial flexibility as detected by SAXS (S4 Fig).

The main core for the folding of nsp1 might be the helix α, which starts to form with the designed fusion construct nsp1(13–50). This helix presents one of the lowest chemical shift deviations (Δδ) when one compares different intermediates with the full-length globular nsp1, and it makes long-range contacts with most of the native β-barrel.

**Fig 9:**
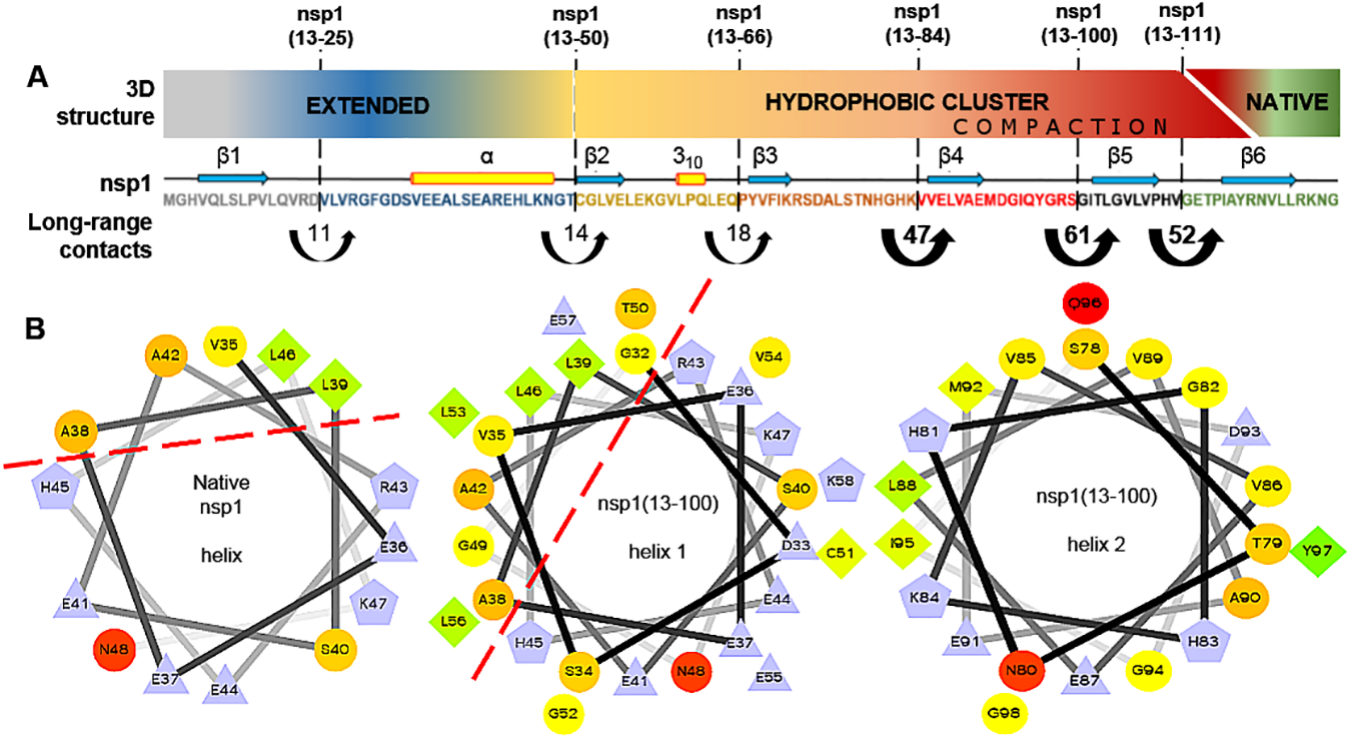
Summary of structural characteristics experimentally determined in the designed fusion proteins. (A) The interpretation of the 3D structure formation is a combination of experimental evidences obtained by bis-ANS fluorescence, SAXS and NMR spectroscopy. The native secondary structure topology is shown above the amino-acid sequence of nsp1, which is colored to distinguish each nsp1 segment used to design the incomplete nsp1 polypeptides. The number of additional long-range contacts between each secondary structure element and the rest of the polypeptide was calculated with MOLMOL on the basis of the native nsp1 3D structure. Thicker arrows and bold number indicate the transitions to more compact 3D structures as identified by SAXS in fusion constructs nsp1(13–100) and nsp1(13–111) and the acquisition of native globular fold in the fusion construct full-length nsp1. (B) Helical topology calculated with the online server rzlab. Dashed lines highlight the interface between hydrophobic and hydrophilic faces of the helices. Helix 1 and 2 were experimentally identified by SSP in the nsp1(13–100) construct (Fig 7).

We summarized the events occurring during the hypothetical folding pathway studied here in Fig 9. The shortest intermediates nsp1(13–25) and nsp1(13–50) are mostly intrinsically disordered, but with residual native α-helix in nsp1(13–50). Subsequently, there is the formation of hydrophobic clusters in nsp1(13–66) with concomitant formation of residual non-native α-helices, which reaches a maximum in nsp1(13–100). The fusion constructs nsp1(13–100) and nsp1(13–111) presents a visible condensation of 3D structure, as detected by SAXS, but only the latter has some stable tertiary structure. A hydrophobic collapse to the native 3D fold might happen only after completion of β-strand 6. The fusion constructs starting from nsp1(13–66), which presents β-strands 1 and 2 and helix α, until nsp1(13–111), resemble molten globules [52]. It is noticeable that the native amphipathic helix α start to be formed within the fusion construct nsp1(13–50) and may provide a template to initiate the tertiary structure of nsp1. A second segment with helical propensity in fusion construct nsp1(13–100) does not show amphipathicity (last panel in Fig 9B), which reinforces the argument that this segment is unstable in this conformation, leaving it with more propensity to form strand β4 and the loops flanking it. Based on our experimental evidence and theoretical analysis, we suggest that nsp1(13–100) represents a crucial step in the cotranslational folding of the β-barrel of nsp1.

## Material and methods

### Recombinant protein production

The plasmid pET25b containing the cDNA encoding the six nsp1 intermediates truncated at different positions at their C-termini and the full-length globular domain C-terminally fused with a 20-amino-acid-linker, followed by the GB1 domain (residues 354–407 of Immunoglobulin G-binding protein G–UniProtKB: P19909) and a HIS-tag, as well as GB1 fused to His-tag, were synthesized by Genescript company. The plasmid containing the full-length globular domain of nsp1 (residues 13–128 of Replicase polyprotein 1ab–UniProtKB: P0C6X7; PDB Id.: 2gdt), consisting of residues 13 to 128, was prepared previously by our group [53]. The truncated constructs are identified in the Table 1.

**Table 1:** Summary of the recombinant constructs of nsp1 used in this study.

Name	Secondary structures	Amino acids	kDa^b^
**nsp1(13–25)**	β1	Gly13—Asp25	11.08
**nsp1(13–50)**	β1-α1	Gly13—Thr50	13.78
**nsp1(13–66)**	β1-α1-β2−3_10_	Gly13—Gln66	15.52
**nsp1(13–84)**	β1-α1-β2−3_10_-β3	Gly13—Lys85	17.57
**nsp1(13–100)**	β1-α1-β2−3_10_-β3-β4	Gly13—Ser100	19.32
**nsp1(13–111)**	β1-α1-β2−3_10_-β3-β4-β5	Gly13—Val111	20.40
**nsp1**^a^	β1-α1-β2−3_10_-β3-β4-β5-β6	Gly13—Gly128	22.19

^a^ the full-length globular domain of nsp1.

^b^ calculated mass of the fusion proteins including the amino-acid sequence of the spacer, GB1 domain and HIS-tag (9.41 kDa).

The plasmids were transformed into the *Escherichia coli* strain BL21 (DE3). Protein expression was achieved by growing the cells in LB (Luria Bertani) medium. The cell culture was shaken at 37°C until an OD_600nm_ of 0.6 was achieved and then induced with 1 mM of isopropyl β-D-1-thiogalactopyranoside (IPTG). Cells were grown for approximately 3 h at the same temperature and harvested by centrifugation. Uniformly ^13^C- and ^15^N-labeled proteins were expressed by growing the cells in M9 minimal medium containing ^15^NH_4_Cl (1 g/L) and (^13^C_6_)-D-glucose (4 g/L) as the sole nitrogen and carbon sources, respectively. Procedures for expression were similar to those used for unlabeled proteins. After expression, the cell cultures were centrifuged at 3000 × *g* for 20 min, at 4°C; the supernatant was discarded.

For protein purification, the cells were lysed by sonication in an ice bath, in the presence of buffer A (50 mM HEPES pH 7.0, 250 mM NaCl, 1 mM dithiothreitol, 3 mM NaN_3_) and protease inhibitors (complete™, EDTA-free from Roche). The debris was removed by centrifugation at 7000 × *g* for 30 min, at 4°C; the supernatant was filtered (0.45 μm) and loaded onto a Ni^2+^ affinity column (HisTrap HP column; GE Healthcare) equilibrated with 90% Buffer A and 10% Buffer B (500 mM imidazole in Buffer A) at 2 mL/min. A linear gradient of 10–100% Buffer B was used to elute the target protein at 4 mL/min. The fractions containing recombinant proteins (determined by SDS-PAGE) were pooled, concentrated using centrifugal filter devices (Vivaspin 20; GE Healthcare^®^) and loaded onto a size-exclusion column (16/60 Superdex™ 75, GE Healthcare^®^) equilibrated with Buffer C (sodium phosphate 50 mM, pH 7.0, 250 mM NaCl,1 mM DTT and 3 mM azide), at 2 mL/min. All samples were in Buffer C for all experimental measurements. Fractions containing the recombinant proteins were concentrated using centrifugal filter devices (Vivaspin 20; GE Healthcare). NMR samples were supplemented with 10% D_2_O. The identity of purified samples was confirmed by MALDI-TOF (data not shown).

### Small angle X-ray scattering

SAXS experiments were performed in the D11-SAXS1 beam line [54] at the National Laboratory for Synchrotron Radiation (LNLS). SAXS data were collected using a two-dimensional detector (Pilatus 300k; Dectris, USA) at a wavelength of 1.548 Å with the sample-detector distance providing a q-range from 0.07 nm^-1^ to 2.5 nm^-1^, where q is the modulus of the scattering vector (calculated according to q = (4π/λ) sinθ, where λ is the wavelength and 2θ is the scattering angle). Three successive frames of 300 sec were collected per sample in order to rule out radiation-induced damage. Frames behaved similarly, and thus we assumed no detectable sample instability during measurements. All three frames were averaged. The data reduction routine was performed with Fit2D [55], including normalization of the one-dimensional scattered data to the intensity of the transmitted incident beam; correction for detector response, incident beam intensity and sample absorption; and blank subtraction using scattering from buffer collected under the same experimental protocol.

The R_g_ and the scattered intensity extrapolated to zero q, I_(q)_, were inferred from the slope and the intercept of the linear fit of ln[I_(q)_] versus q^2^ in the q-range q×Rg < 1.3 [56] and also computed from the indirect Fourier transform program Gnom [57]. From these data we inferred the monodispersity of all protein constructs. We also used Gnom to compute the distance-distribution function, P_(r)_, its R_g_ and the maximum dimension, D_max_.

### Nuclear magnetic resonance spectroscopy

NMR spectra were collected at 22°C with Bruker Ascend 500 MHz, Ascend 700 MHz, and Avance 800 MHz spectrometers equipped with z axis gradient 5-mm triple gradient probes and Avance 600-MHz, equipped with a z axis gradient 5-mm triple resonance cryogenic probe. 2D [^1^H,^15^N]-HSQC, 3D HNCO, 3D HNCACB, 3D CBCA(CO)NH, 3D HBHA(CO)NH, and 3D ^15^N-edited [^1^H,^1^H] NOESY spectra [58] were used to obtain sequence-specific assignments for the polypeptide backbone of nsp1(13–25), nsp1(13–50) and nsp1(13–100) constructs in Buffer C. For the resonance assignments of GB1 solubility domain we followed the J-UNIO protocol [59]. Briefly, we performed automated backbone assignment using 5D APSY-CBCACONH, 4D APSY-HACANH and 5D APSY-HACACONH data sets [60,61] as input for the software UNIO-MATCH [62], which yielded 98% of the expected chemical shifts after interactive validation using the 3D ^15^N-edited [^1^H,^1^H] NOESY spectra. The chemical shift assignments for nsp1 globular domain have been published elsewhere [63].

Steady-state ^15^N{^1^H} NOEs [64,65] were measured on a Bruker Avance 800-MHz spectrometer, using a saturation period of 3 s and an interscan delay of 5 s. The errors in the primary intensity data were taken from the root-mean-square noise of background regions in the spectra [66].

The secondary structure propensity was calculated using the algorithm SSP [67]. The C^α^, C^β^ and H^α^ chemical shifts of nsp1(13–25), nsp1(13–50) and nsp1(13–100) constructs, as well as GB1 and nsp1 globular domains, were used as input to calculate the propensity to form α-helix (SSP > 0), or extended conformation such as in β-strands (SSP < 0).

The ^1^H chemical shifts were referenced to internal sodium-3-(trimethylsilyl) propanesulfonate (DSS). Using the absolute frequency ratios (0.251449530 and 0.101329118), the ^13^C and ^15^N chemical shifts were referenced indirectly to DSS [68,69]. The NMR spectra were processed with Topspin 3 and analyzed with CARA 1.9.1.5 [70]. The NMR chemical shifts of the protein constructs have been deposited in the BioMagResBank under BMRB codes 27169, 27176, 27177, and 27178.

### Circular dichroism spectroscopy

CD experiments were carried out using a Chirascan^TM^, CD Spectrometer (Applied Photophysics) with a 0.1-cm path length quartz cuvette. CD spectra were recorded using 30 μM protein in Buffer C. Far-UV spectra were recorded from 190 to 260 nm, averaged over three scans at a speed of 0.5 nm/min, and collected in steps of 0.5 nm. The buffer baselines were automatically subtracted from the respective sample spectra. The raw data were processed using the software ProView, provided by the manufacturer. CD data was reported as mean residue molar ellipicity (deg×cm^2^×dmol^-1^).

### Bis-ANS extrinsic fluorescence experiments

The compactness of the protein hydrophobic cores was assessed using bis-ANS (4,40-dianilino-1,10-binaphthyl-5,50-disulfonic acid) fluorescence spectroscopy. The fluorescence emission spectra of bis-ANS were recorded from 400 to 600 nm using an excitation wavelength of 360 nm, after 5 minutes of incubation of the fluorescent die with each protein. The experiments were conducted with 30 μM protein plus 30 μM bis-ANS, in buffer C. Spectra of bis-ANS without protein and protein without bis-ANS were used as controls. All of the fluorescence experiments were recorded using a VARIAN Cary ECLIPSE^®^ fluorimeter (Palo Alto, CA) at 22°C, and the data analyzed with GraphPad Prism 6.01.

## Supporting information

S1 Fig Comparison of the NMR chemical shifts of GB1 domains. The GB1 domain and the fusion constructs nsp1(13–25), nsp1(13–50) and nsp1(13–100) had their NMR chemical shifts assigned and then pairwise compared among each protein, as a probe for 3D structure comparison. (A) Combined chemical shift differences of ^1^HN and ^15^N, between the GB1 domains fused to His-tag or to the designed fusion protein nsp1(13–25). (B) Cartoon of the GB1 domain polypeptide backbone fold highlighting in orange the locations of residues that exhibit a combined chemical shift difference greater than one standard deviation above the average (Δδ = 0.16 ppm) and in blue for residues that exhibited chemical shift difference greater than the average (Δδ = 0.06 ppm). (C) Combined chemical shift differences of ^1^HN and ^15^N, between the GB1 domains in the designed fusion proteins nsp1(13–25) and nsp1(13–50), or nsp1(13–50) and nsp1(13–100).
(TIF)

S2 Fig NMR analysis of the linker and GB1 solubility domain in the designed fusion protein nsp1(13–25). (A) 2D [^1^H,^15^N]-HSQC of the designed fusion protein nsp1(13–25). The orange crosses identify the HN backbone signals of the spacer polypeptide. (B) Secondary structure propensity of the spacer and GB1 domain. Positive values identify propensity to adopt helix while negative values identify propensity to form extended polypeptide structures such as β-strands. (C) ^15^N{^1^H}-NOEs of the spacer and GB1 domain. Positive values represent less dynamic to rigid residues, while close-to-zero and negative values identify highly dynamic residues. The standard errors are indicated. The hatched bars identify positions without data.
(TIF)

S3 Fig Diagram highlighting the long-range contacts of native nsp1 domain. The blue shapes represent each secondary structure element. The total number of long-range contacts to each segment is indicated in white labels. Long-range contacts among β-strands are indicated by both a line and a number on that line, and the contacts of a β-strand with the helix α is represented by a line and the numbers inside the shape that represents the helix. The long-range contacts were measured with MOLMOL and represent atoms that are at most 2.4 Å of distance and between at least 4 residues away.
(TIF)

S4 Fig SAXS analysis of the designed fusion proteins and GB1 domain. (A) Scattered intensities. (B) Kratky plot of the scattering curves.
(TIF)

## References

[1] Kihara HK, Hu ASL, Halvorson HO. THE IDENTIFICATION OF A RIBOSOMAL-BOUND β-GLUCOSIDASE*. Proc Natl Acad Sci U S A. 1961;47: 489–497. 13755892 10.1073/pnas.47.4.489 PMC221478

[2] Friguet B, Djavadi-Ohaniance L, King J, Goldberg ME. In vitro and ribosome-bound folding intermediates of P22 tailspike protein detected with monoclonal antibodies. J Biol Chem. 1994;269: 15945–15949. 7515066

[3] Hamlin J, Zabin I. β-Galactosidase: Immunological Activity of Ribosome-Bound, Growing Polypeptide Chains. Proc Natl Acad Sci U S A. 1972;69: 412–416. 4551143 10.1073/pnas.69.2.412 PMC426469

[4] De Prat Gay G, Ruiz-Sanz J, Neira JL, Itzhaki LS, Fersht AR. Folding of a nascent polypeptide chain in vitro: cooperative formation of structure in a protein module. Proc Natl Acad Sci U S A. 1995;92: 3683–3686. 7731965 10.1073/pnas.92.9.3683 PMC42025

[5] de Prat Gay G, Ruiz-Sanz J, Neira JL, Corrales FJ, Otzen DE, Ladurner AG, et al Conformational Pathway of the Polypeptide Chain of Chymotrypsin Inhibitor-2 Growing from its N Terminusin vitro. Parallels with the Protein Folding Pathway. J Mol Biol. 1995;254: 968–979. doi: 10.1006/jmbi.1995.0669 7500364 10.1006/jmbi.1995.0669

[6] Ugrinov KG, Clark PL. Cotranslational Folding Increases GFP Folding Yield. Biophys J. 2010;98: 1312–1320. doi: 10.1016/j.bpj.2009.12.4291 20371331 10.1016/j.bpj.2009.12.4291 PMC2849091

[7] Kelkar DA, Khushoo A, Yang Z, Skach WR. Kinetic Analysis of Ribosome-bound Fluorescent Proteins Reveals an Early, Stable, Cotranslational Folding Intermediate. J Biol Chem. 2012;287: 2568–2578. doi: 10.1074/jbc.M111.318766 22128180 10.1074/jbc.M111.318766 PMC3268416

[8] Kim SJ, Yoon JS, Shishido H, Yang Z, Rooney LA, Barral JM, et al Translational tuning optimizes nascent protein folding in cells. Science. 2015;348: 444–448. doi: 10.1126/science.aaa3974 25908822 10.1126/science.aaa3974

[9] Zhang G, Hubalewska M, Ignatova Z. Transient ribosomal attenuation coordinates protein synthesis and co-translational folding. Nat Struct Mol Biol. 2009;16: 274–280. doi: 10.1038/nsmb.1554 19198590 10.1038/nsmb.1554

[10] Komar AA. A pause for thought along the co-translational folding pathway. Trends Biochem Sci. 2009;34: 16–24. doi: 10.1016/j.tibs.2008.10.002 18996013 10.1016/j.tibs.2008.10.002

[11] Thanaraj TA, Argos P. Ribosome-mediated translational pause and protein domain organization. Protein Sci. 1996;5: 1594–1612. doi: 10.1002/pro.5560050814 8844849 10.1002/pro.5560050814 PMC2143486

[12] Buhr F, Jha S, Thommen M, Mittelstaet J, Kutz F, Schwalbe H, et al Synonymous Codons Direct Cotranslational Folding toward Different Protein Conformations. Mol Cell. 2016;61: 341–351. doi: 10.1016/j.molcel.2016.01.008 26849192 10.1016/j.molcel.2016.01.008 PMC4745992

[13] Tsai C-J, Sauna ZE, Kimchi-Sarfaty C, Ambudkar SV, Gottesman MM, Nussinov R. Synonymous Mutations and Ribosome Stalling Can Lead to Altered Folding Pathways and Distinct Minima. J Mol Biol. 2008;383: 281–291. doi: 10.1016/j.jmb.2008.08.012 18722384 10.1016/j.jmb.2008.08.012 PMC2628389

[14] Siller E, DeZwaan DC, Anderson JF, Freeman BC, Barral JM. Slowing Bacterial Translation Speed Enhances Eukaryotic Protein Folding Efficiency. J Mol Biol. 2010;396: 1310–1318. doi: 10.1016/j.jmb.2009.12.042 20043920 10.1016/j.jmb.2009.12.042

[15] Gay GDP, Ruiz-Sanz J, Neira JL, Itzhaki LS, Fersht AR. Folding of a nascent polypeptide chain in vitro: cooperative formation of structure in a protein module. Proc Natl Acad Sci. 1995;92: 3683–3686. 7731965 10.1073/pnas.92.9.3683 PMC42025

[16] de Prat Gay G, Ruiz-Sanz J, Neira JL, Corrales FJ, Otzen DE, Ladurner AG, et al Conformational pathway of the polypeptide chain of chymotrypsin inhibitor-2 growing from its N terminusin vitro. Parallels with the protein folding pathway. J Mol Biol. 1995;254: 968–979. doi: 10.1006/jmbi.1995.0669 7500364 10.1006/jmbi.1995.0669

[17] Cabrita LD, Cassaignau AME, Launay HMM, Waudby CA, Wlodarski T, Camilloni C, et al A structural ensemble of a ribosome–nascent chain complex during cotranslational protein folding. Nat Struct Mol Biol. 2016;23: 278–285. doi: 10.1038/nsmb.3182 26926436 10.1038/nsmb.3182 PMC5405865

[18] Samelson AJ, Jensen MK, Soto RA, Cate JHD, Marqusee S. Quantitative determination of ribosome nascent chain stability. Proc Natl Acad Sci. 2016;113: 13402–13407. doi: 10.1073/pnas.1610272113 27821780 10.1073/pnas.1610272113 PMC5127326

[19] Nilsson OB, Hedman R, Marino J, Wickles S, Bischoff L, Johansson M, et al Cotranslational Protein Folding inside the Ribosome Exit Tunnel. Cell Rep. 2015;12: 1533–1540. doi: 10.1016/j.celrep.2015.07.065 26321634 10.1016/j.celrep.2015.07.065 PMC4571824

[20] Waudby CA, Launay H, Cabrita LD, Christodoulou J. Protein folding on the ribosome studied using NMR spectroscopy. Prog Nucl Magn Reson Spectrosc. 2013;74: 57–75. doi: 10.1016/j.pnmrs.2013.07.003 24083462 10.1016/j.pnmrs.2013.07.003 PMC3991860

[21] Kaiser CM, Goldman DH, Chodera JD, Tinoco I, Bustamante C. The Ribosome Modulates Nascent Protein Folding. Science. 2011;334: 1723–1727. doi: 10.1126/science.1209740 22194581 10.1126/science.1209740 PMC4172366

[22] O’Brien EP, Hsu S-TD, Christodoulou J, Vendruscolo M, Dobson CM. Transient Tertiary Structure Formation within the Ribosome Exit Port. J Am Chem Soc. 2010;132: 16928–16937. doi: 10.1021/ja106530y 21062068 10.1021/ja106530y

[23] Kosolapov A, Deutsch C. Tertiary interactions within the ribosomal exit tunnel. Nat Struct Mol Biol. 2009;16: 405–411. doi: 10.1038/nsmb.1571 19270700 10.1038/nsmb.1571 PMC2670549

[24] Huth JR, Bewley CA, Clore GM, Gronenborn AM, Jackson BM, Hinnebusch AG. Design of an expression system for detecting folded protein domains and mapping macromolecular interactions by NMR. Protein Sci. 2008;6: 2359–2364. doi: 10.1002/pro.5560061109 9385638 10.1002/pro.5560061109 PMC2143577

[25] Kuszewski J, Clore GM, Gronenborn AM. Fast folding of a prototypic polypeptide: The immunoglobulin binding domain of streptococcal protein G. Protein Sci. 1994;3: 1945–1952. doi: 10.1002/pro.5560031106 7703841 10.1002/pro.5560031106 PMC2142643

[26] Williamson RA, Carr MD, Frenkiel TA, Feeney J, Freedman RB. Mapping the Binding Site for Matrix Metalloproteinase on the N-Terminal Domain of the Tissue Inhibitor of Metalloproteinases-2 by NMR Chemical Shift Perturbation ^†^. Biochemistry (Mosc). 1997;36: 13882–13889. doi: 10.1021/bi9712091 9374866 10.1021/bi9712091

[27] Williamson MP. Using chemical shift perturbation to characterise ligand binding. Prog Nucl Magn Reson Spectrosc. 2013;73: 1–16. doi: 10.1016/j.pnmrs.2013.02.001 23962882 10.1016/j.pnmrs.2013.02.001

[28] van Montfort RLM, Bateman OA, Lubsen NH, Slingsby C. Crystal structure of truncated human βB1-crystallin. Protein Sci. 2009;12: 2606–2612. doi: 10.1110/ps.03265903 14573871 10.1110/ps.03265903 PMC2366963

[29] Bax B, Lapatto R, Nalini V, Driessen H, Lindley PF, Mahadevan D, et al X-ray analysis of βB2-crystallin and evolution of oligomeric lens proteins. Nature. 1990;347: 776–780. doi: 10.1038/347776a0 2234050 10.1038/347776a0

[30] Kelly S, Price N. The Use of Circular Dichroism in the Investigation of Protein Structure and Function. Curr Protein Pept Sci. 2000;1: 349–384. doi: 10.2174/1389203003381315 12369905 10.2174/1389203003381315

[31] Rosen CG, Weber G. Dimer formation from 1-anilino-8-naphthalenesulfonate catalyzed by bovine serum albumin. Fluorescent molecule with exceptional binding properties. Biochemistry (Mosc). 1969;8: 3915–3920. doi: 10.1021/bi00838a006 10.1021/bi00838a006 5388144

[32] Hawe A, Sutter M, Jiskoot W. Extrinsic Fluorescent Dyes as Tools for Protein Characterization. Pharm Res. 2008;25: 1487–1499. doi: 10.1007/s11095-007-9516-9 18172579 10.1007/s11095-007-9516-9 PMC2440933

[33] Black SD, Mould DR. Development of hydrophobicity parameters to analyze proteins which bear post- or cotranslational modifications. Anal Biochem. 1991;193: 72–82. 2042744 10.1016/0003-2697(91)90045-u

[34] Agashe VR, Shastry MCR, Udgaonkar JB. Initial hydrophobic collapse in the folding of barstar. Nature. 1995;377: 754–757. doi: 10.1038/377754a0 7477269 10.1038/377754a0

[35] Crist B. A review of: “SMALL ANGLE X-RAY SCATTERING”, edited by O. Glatter and O. Kratky (Universitat Graz, Austria) New York: Academic Press, 1982, 515 pp. $89.50. ISBN 0-12-286280. Chem Eng Commun. 1983;22: 377–378. doi:10.1080/00986448308940069

[36] Mertens HDT, Svergun DI. Structural characterization of proteins and complexes using small-angle X-ray solution scattering. J Struct Biol. 2010;172: 128–141. doi: 10.1016/j.jsb.2010.06.012 20558299 10.1016/j.jsb.2010.06.012

[37] Wilkins DK, Grimshaw SB, Receveur V, Dobson CM, Jones JA, Smith LJ. Hydrodynamic Radii of Native and Denatured Proteins Measured by Pulse Field Gradient NMR Techniques ^†^. Biochemistry (Mosc). 1999;38: 16424–16431. doi: 10.1021/bi991765q 10.1021/bi991765q 10600103

[38] Wüthrich K, Wagner G. Internal motion in globular proteins. Trends Biochem Sci. 1978;3: 227–230. doi: 10.1016/S0968-0004(78)94607-8

[39] Wishart D, Sykes B. The 13C Chemical-Shift Index: A simple method for the identification of protein secondary structure using 13C chemical-shift data. J Biomol NMR. 1994;4 doi: 10.1007/BF00175245 10.1007/BF00175245 8019132

[40] Wishart DS, Sykes BD, Richards FM. The chemical shift index: a fast and simple method for the assignment of protein secondary structure through NMR spectroscopy. Biochemistry (Mosc). 1992;31: 1647–1651. 10.1021/bi00121a010 1737021

[41] Marsh JA, Singh VK, Jia Z, Forman-Kay JD. Sensitivity of secondary structure propensities to sequence differences between α- and γ-synuclein: Implications for fibrillation. Protein Sci. 2006;15: 2795–2804. doi: 10.1110/ps.062465306 17088319 10.1110/ps.062465306 PMC2242444

[42] Chow CC, Chow C, Raghunathan V, Huppert TJ, Kimball EB, Cavagnero S. Chain Length Dependence of Apomyoglobin Folding: Structural Evolution from Misfolded Sheets to Native Helices ^†^. Biochemistry (Mosc). 2003;42: 7090–7099. doi: 10.1021/bi0273056 12795605 10.1021/bi0273056

[43] Kay LE, Torchia DA, Bax A. Backbone dynamics of proteins as studied by 15N inverse detected heteronuclear NMR spectroscopy: application to staphylococcal nuclease. Biochemistry (Mosc). 1989;28: 8972–8979. 10.1021/bi00449a003 2690953

[44] Kuwajima K, Yamaya H, Miwa S, Sugai S, Nagamura T. Rapid formation of secondary structure framework in protein folding studied by stopped-flow circular dichroism. FEBS Lett. 1987;221: 115–118. doi: 10.1016/0014-5793(87)80363-0 3040467 10.1016/0014-5793(87)80363-0

[45] Watanabe M, Kobashigawa Y, Aizawa T, Demura M, Nitta K. A Non-Native α-Helix Is Formed in the -Sheet Region of the Molten Globule State of Canine Milk Lysozyme. Protein J. 2004;23: 335–342. doi: 10.1023/B:JOPC.0000032653.30096.41 15328889 10.1023/b:jopc.0000032653.30096.41

[46] Ikeguchi M. Transient Non-Native Helix Formation during the Folding of b-Lactoglobulin. Biomolecules. 2014;4: 202–216. doi: 10.3390/biom4010202 24970212 10.3390/biom4010202 PMC4030977

[47] Non-native alpha-helical intermediate in the refolding of beta-lactoglobulin, a predominantly beta-sheet protein.—PubMed—NCBI [Internet]. [cited 13 Apr 2017]. Available: https://www.ncbi.nlm.nih.gov/pubmed/8836104 10.1038/nsb1096-868 8836104

[48] Chow CC, Chow C, Raghunathan V, Huppert TJ, Kimball EB, Cavagnero S. Chain length dependence of apomyoglobin folding: structural evolution from misfolded sheets to native helices. Biochemistry (Mosc). 2003;42: 7090–7099. 10.1021/bi0273056 12795605

[49] Matouschek A, Kellis JT, Serrano L, Bycroft M, Fersht AR. Transient folding intermediates characterized by protein engineering. Nature. 1990;346: 440–445. doi: 10.1038/346440a0 2377205 10.1038/346440a0

[50] Flanagan JM, Kataoka M, Shortle D, Engelman DM. Truncated staphylococcal nuclease is compact but disordered. Proc Natl Acad Sci. 1992;89: 748–752. 1731350 10.1073/pnas.89.2.748 PMC48316

[51] Neira JL, Fersht AR. Exploring the folding funnel of a polypeptide chain by biophysical studies on protein fragments. J Mol Biol. 1999;285: 1309–1333. doi: 10.1006/jmbi.1998.2249 9887278 10.1006/jmbi.1998.2249

[52] Bose HS, Whittal RM, Baldwin MA, Miller WL. The active form of the steroidogenic acute regulatory protein, StAR, appears to be a molten globule. Proc Natl Acad Sci. 1999;96: 7250–7255. doi: 10.1073/pnas.96.13.7250 10377400 10.1073/pnas.96.13.7250 PMC22068

[53] Almeida MS, Johnson MA, Herrmann T, Geralt M, Wüthrich K. Novel β-barrel fold in the nuclear magnetic resonance structure of the replicase nonstructural protein 1 from the severe acute respiratory syndrome coronavirus. J Virol. 2007;81: 3151–3161. doi: 10.1128/JVI.01939-06 17202208 10.1128/JVI.01939-06 PMC1866046

[54] Kellermann G, Vicentin F, Tamura E, Rocha M, Tolentino H, Barbosa A, et al The small-angle X-ray scattering beamline of the Brazilian Synchrotron Light Laboratory. J Appl Crystallogr. 1997;30: 880–883.

[55] Hammersley AP. FIT2D: an introduction and overview. Eur Synchrotron Radiat Facil Intern Rep ESRF97HA02T. 1997;68 Available: http://128.40.77.181/ccp/ccp14/ftp-mirror/fit2d/ftp.esrf.fr/pub/expg/FIT2D/fit2d_intro.ps

[56] Guimer A, Fournet G. Small angle scattering of X-rays. J Wiley Sons N Y. 1955; Available: http://www.eng.uc.edu/~beaucag/Classes/Scattering/GuinierandFournet.pdf

[57] Svergun DI. Determination of the regularization parameter in indirect-transform methods using perceptual criteria. J Appl Crystallogr. 1992;25: 495–503.

[58] Bax AD, Grzesiek S. Methodological advances in protein NMR NMR of Proteins. Springer; 1993 pp. 33–52. Available: http://link.springer.com/chapter/10.1007/978-1-349-12749-8_2

[59] Serrano P, Pedrini B, Mohanty B, Geralt M, Herrmann T, Wüthrich K. The J-UNIO protocol for automated protein structure determination by NMR in solution. J Biomol NMR. 2012;53: 341–354. doi: 10.1007/s10858-012-9645-2 22752932 10.1007/s10858-012-9645-2 PMC3541938

[60] Hiller S, Fiorito F, Wüthrich K, Wider G. Automated projection spectroscopy (APSY). Proc Natl Acad Sci U S A. 2005;102: 10876–10881. doi: 10.1073/pnas.0504818102 16043707 10.1073/pnas.0504818102 PMC1182451

[61] Hiller S, Wider G, Wüthrich K. APSY-NMR with proteins: practical aspects and backbone assignment. J Biomol NMR. 2008;42: 179–195. doi: 10.1007/s10858-008-9266-y 18841481 10.1007/s10858-008-9266-y

[62] Volk J, Herrmann T, Wüthrich K. Automated sequence-specific protein NMR assignment using the memetic algorithm MATCH. J Biomol NMR. 2008;41: 127–138. doi: 10.1007/s10858-008-9243-5 18512031 10.1007/s10858-008-9243-5

[63] Almeida MS, Johnson MA, Wüthrich K. NMR assignment of the SARS-CoV protein nsp1. J Biomol NMR. 2006;36: 46–46. doi: 10.1007/s10858-006-9018-9 16821128 10.1007/s10858-006-9018-9 PMC7087757

[64] Zhu G, Xia Y, Nicholson LK, Sze KH. Protein dynamics measurements by TROSY-based NMR experiments. J Magn Reson. 2000;143: 423–426. doi: 10.1006/jmre.2000.2022 10729271 10.1006/jmre.2000.2022

[65] Renner C, Schleicher M, Moroder L, Holak TA. Practical aspects of the 2D 15N-${$1H$}$-NOE experiment. J Biomol NMR. 2002;23: 23–33. 12061715 10.1023/a:1015385910220

[66] Kay LE, Torchia DA, Bax A. Backbone dynamics of proteins as studied by nitrogen-15 inverse detected heteronuclear NMR spectroscopy: application to staphylococcal nuclease. Biochemistry (Mosc). 1989;28: 8972–8979. 10.1021/bi00449a003 2690953

[67] Marsh JA, Singh VK, Jia Z, Forman-Kay JD. Sensitivity of secondary structure propensities to sequence differences between α-and γ-synuclein: Implications for fibrillation. Protein Sci. 2006;15: 2795–2804. doi: 10.1110/ps.062465306 17088319 10.1110/ps.062465306 PMC2242444

[68] Wishart DS, Bigam CG, Yao J, Abildgaard F, Dyson HJ, Oldfield E, et al 1H, 13C and 15N chemical shift referencing in biomolecular NMR. J Biomol NMR. 1995;6: 135–140. 8589602 10.1007/BF00211777

[69] Markley JL, Bax A, Arata Y, Hilbers CW, Kaptein R, Sykes BD, et al Recommendations for the presentation of NMR structures of proteins and nucleic acids. J Mol Biol. 1998;280: 933–952. doi: 10.1006/jmbi.1998.1852 9671561 10.1006/jmbi.1998.1852

[70] Keller R, Wuthrich K. Computer-aided resonance assignment (CARA). Verl Goldau Cantina Switz. 2004;

