# Supplementary figures and images for: Comprehensive structural analysis of designed incomplete polypeptide chains of the replicase nonstructural protein 1 from the severe acute respiratory syndrome coronavirus
Source: PLoS One. 2017 Jul 27;12(7):e0182132. doi: 10.1371/journal.pone.0182132 (PMC5531528; doi:10.1371/journal.pone.0182132)

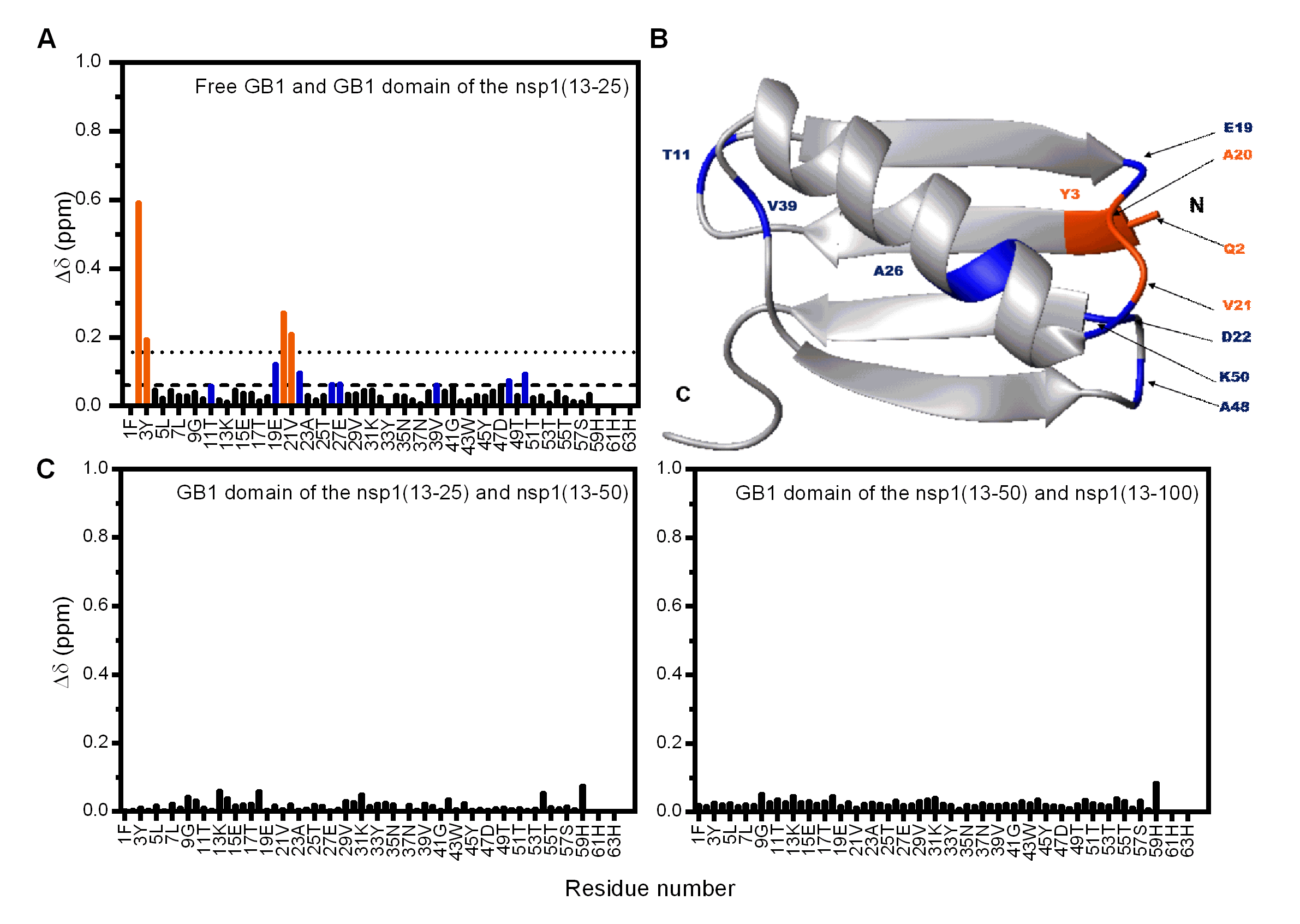

Supplement: S1 Fig — The GB1 domain and the fusion constructs nsp1(13–25), nsp1(13–50) and nsp1(13–100) had their NMR chemical shifts assigned and then pairwise compared among each protein, as a probe for 3D structure comparison. (A) Combined chemical shift differences of 1HN and 15N, between the GB1 domains fused to His-tag or to the designed fusion protein nsp1(13–25). (B) Cartoon of the GB1 domain polypeptide backbone fold highlighting in orange the locations of residues that exhibit a combined chemical shift difference greater than one standard deviation above the average (Δδ = 0.16 ppm) and in blue for residues that exhibited chemical shift difference greater than the average (Δδ = 0.06 ppm). (C) Combined chemical shift differences of 1HN and 15N, between the GB1 domains in the designed fusion proteins nsp1(13–25) and nsp1(13–50), or nsp1(13–50) and nsp1(13–100). (TIF) [file pone.0182132.s001.tif]

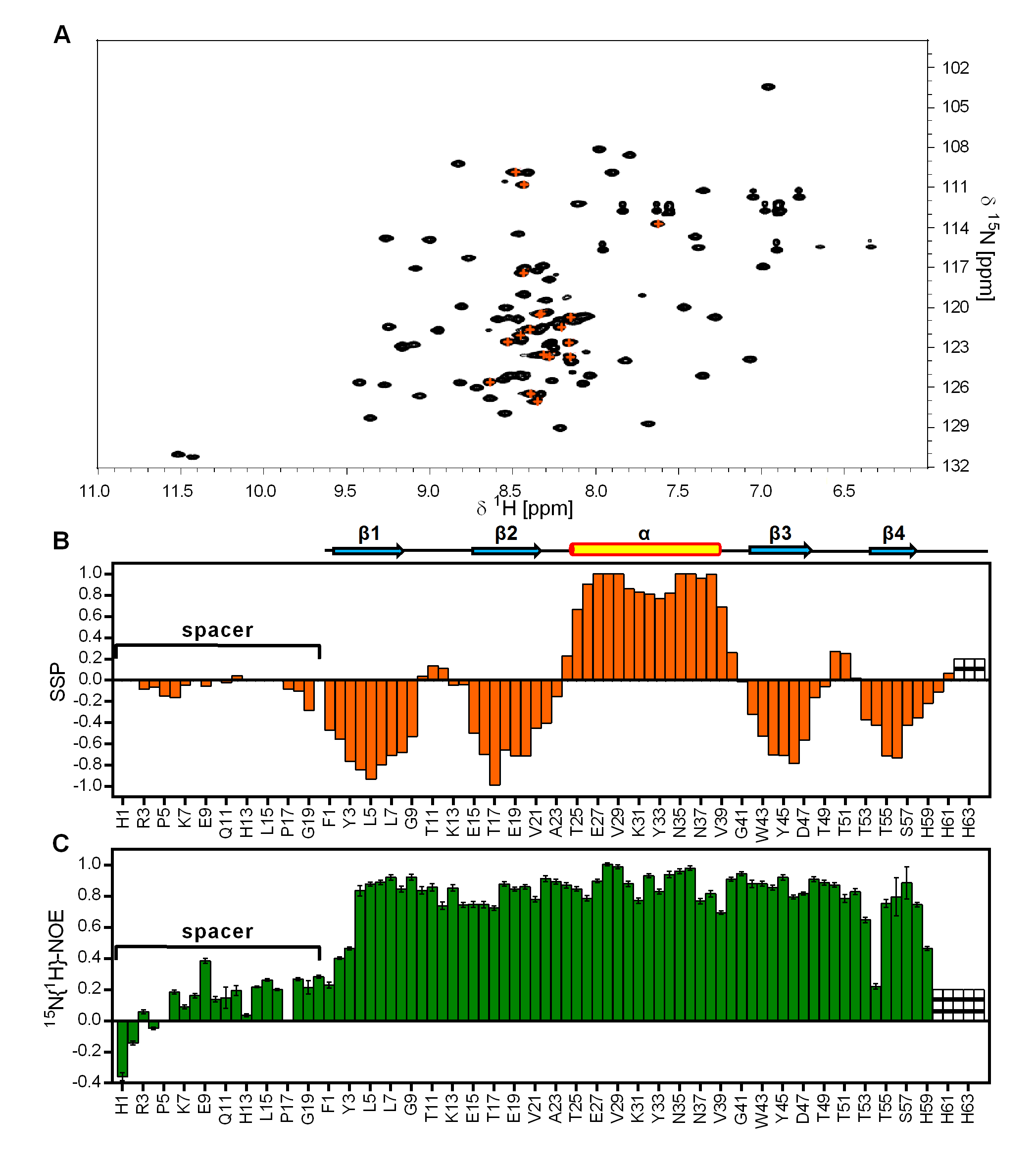

Supplement: S2 Fig — (A) 2D [1H,15N]-HSQC of the designed fusion protein nsp1(13–25). The orange crosses identify the HN backbone signals of the spacer polypeptide. (B) Secondary structure propensity of the spacer and GB1 domain. Positive values identify propensity to adopt helix while negative values identify propensity to form extended polypeptide structures such as β-strands. (C) 15N{1H}-NOEs of the spacer and GB1 domain. Positive values represent less dynamic to rigid residues, while close-to-zero and negative values identify highly dynamic residues. The standard errors are indicated. The hatched bars identify positions without data. (TIF) [file pone.0182132.s002.tif]

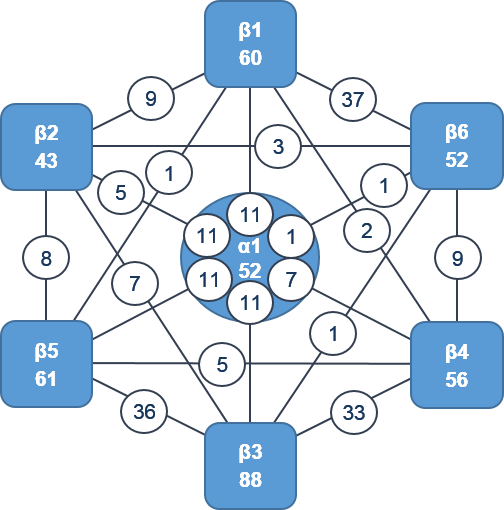

Supplement: S3 Fig — The blue shapes represent each secondary structure element. The total number of long-range contacts to each segment is indicated in white labels. Long-range contacts among β-strands are indicated by both a line and a number on that line, and the contacts of a β-strand with the helix α is represented by a line and the numbers inside the shape that represents the helix. The long-range contacts were measured with MOLMOL and represent atoms that are at most 2.4 Å of distance and between at least 4 residues away. (TIF) [file pone.0182132.s003.tif]

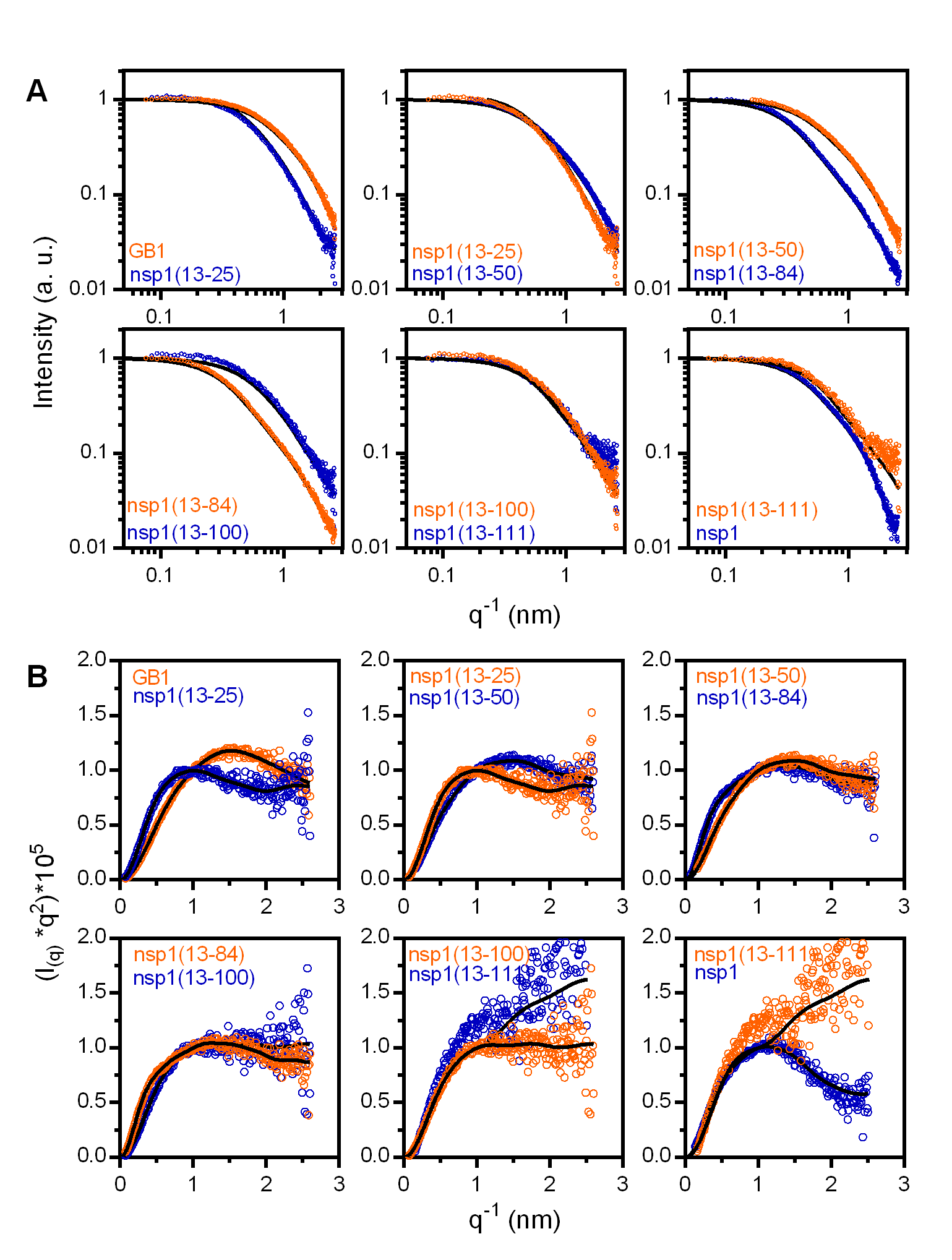

Supplement: S4 Fig — (A) Scattered intensities. (B) Kratky plot of the scattering curves. (TIF) [file pone.0182132.s004.tif]
